# Acetylation in renal physiology and pathophysiology

**DOI:** 10.3389/fphar.2025.1660109

**Published:** 2025-10-01

**Authors:** Jian-Yu Zhang, Jun Wu, Zi-Han Chen, Shi-Yue Liu, Ping Li, Dan-Qian Chen

**Affiliations:** ^1^ School of Life Science, Northwest University, Xi’an, Shaanxi, China; ^2^ Office of Research Administration, Shandong College of Traditional Chinese Medicine, Yantai, Shandong, China; ^3^ Beijing Key Lab for Immune-Mediated Inflammatory Diseases, Institute of Clinical Medical Sciences, China-Japan Friendship Hospital, Beijing, China

**Keywords:** Acetylation, AKI, CKD, HDACs, SIRTs

## Abstract

The kidney, one of the most important organs in the human body, is vital for maintaining overall health and homeostasis. However, kidney diseases, including acute kidney injury (AKI) and chronic kidney disease (CKD), have become serious global public health issues. Post-translational modification (PTM) of proteins, especially acetylation, can affect the pathophysiology of the kidney through various pathways, including the regulation of inflammatory responses, fibrosis, apoptosis, and autophagy. Acetylation is primarily regulated by two enzymes: histone acetyltransferases (HATs) and histone deacetylases (HDACs). There are 11 known HDAC isoforms that influence the onset and progression of kidney disease by affecting the acetylation level of key proteins. Additionally, sirtuins (SIRTs), which belonging to class III HDACs, regulate multiple biological processes to exert protective effects on the kidneys and delay the progression of kidney diseases. Intriguingly, some SIRTs exhibit dual roles (protective/detrimental) in various renal disease models. Many HDAC inhibitors and SIRT activators have been widely used in the clinical treatment of various kidney diseases. In this review, we summarize the roles and mechanisms of HDACs and SIRTs in kidney diseases and then review the potential therapeutic effects of some SIRT activators and HDAC inhibitors in kidney protection. Notably, we also discuss the mechanism of SIRTs with dual roles in kidney protection and injury and introduce some agonists and inhibitors targeting these SIRTs.

## 1 Introduction

The kidney, a vital organ responsible for filtration, reabsorption, and regulation of various physiological functions, maintains internal environmental homeostasis (Silva-Aguiar et al., 2022). However, kidney diseases, including acute kidney injury (AKI) and chronic kidney disease (CKD), impose a substantial burden on global healthcare systems due to their high mortality rates and the limited treatment options (Chade and Bidwell, 2022).

In recent years, the role of post-translation modification (PTM) in renal diseases has received widespread attention. PTM has the ability to alter the biological activity, function, and location of multiple proteins and is even involved in the pathogenesis of many renal diseases (Wu X. et al., 2023; Hou et al., 2024). PTM exerts a variety of effects on kidney diseases, which can be either protective or detrimental, depending on the target proteins and the specific type of protein PTM. Different PTMs influence various signaling pathways during kidney injury, thus contributing to new strategies and potential therapeutic targets for kidney diseases (Liu Z. et al., 2023). Lysine acetylation is a common PTM process of proteins, which plays an important role in renal physiology and pathology and affects the physiological homeostasis of the kidney. It is synergistically regulated by two enzymes with contrasting functions: lysine acetyltransferase catalyzes the addition of acetyl groups, whereas lysine deacetylase mediates the removal of acetyl groups, and these two enzymes act on both histone and non-histone proteins (Shvedunova and Akhtar, 2022; Li X. Y. et al., 2023).

Histone acetyltransferases (HATs) and histone deacetylases (HDACs) can significantly influence critical physiological processes including development, metabolic homeostasis, immune function, inflammatory responses, oxidative stress, and autophagy by regulating gene expression, cell proliferation and differentiation, and cell survival, thereby influencing the initiation and progression of kidney diseases (Liu Y. et al., 2021; Wang et al., 2023b; Wu F. et al., 2023). Basal autophagy in renal cells plays a crucial role in maintaining renal homeostasis, structure, and function. Autophagy dysfunction contributes to the pathogenesis of AKI, incomplete renal repair after AKI, and CKD (Tang et al., 2020). In addition, autophagy plays a key role in the occurrence and development of diabetic kidney disease (DKD). Abnormal autophagy function of podocytes can cause podocyte damage, even leading to their apoptosis and shedding, which in turn disrupts the structural integrity of the glomerular filtration barrier and ultimately results in proteinuria (Ruan Z. et al., 2024). It is worth noting that HDACs can regulate the related signaling pathways such as phosphatidylinositol-3-kinase (PI3K)/protein kinase B (PKB/Akt) by affecting protein acetylation levels. This effect can significantly regulate the production of key inflammatory mediators and autophagy, thereby influencing inflammatory responses and cellular homeostasis (Jeon et al., 2022; Lee et al., 2022; Lu Y. et al., 2024; Jasim et al., 2025). Aging is an important risk factor for the occurrence and progression of kidney diseases. During this process, cells will produce an imbalance between reactive oxygen species (ROS) generation and the antioxidant defense system, thereby triggering oxidative stress and ultimately leading to kidney damage. However, HDACs can delay the progression of aging-related kidney diseases and other aging-related diseases by regulating oxidative stress responses (Ogura et al., 2021).

HATs and HDACs coordinately regulate the histone acetylation status, a process crucial for maintaining normal kidney physiology and influencing the onset and progression of kidney diseases (Shvedunova and Akhtar, 2022). HDACs are classified into four categories: classes I, II, and IV HDACs are Zn^2+^-dependent enzymes, whereas class III HDACs are NAD^+^-dependent deacetylases (Hyndman, 2020). Class II HDACs are further subdivided into two categories: Class IIa, which includes HDAC4, HDAC5, HDAC7, and HDAC9, and Class IIb, which includes HDAC6 and HDAC10. Class III HDACs form a large family, encompassing SIRT1–SIRT7. The sole member of Class IV HDAC is HDAC11 (Shen and Zhuang, 2022b). Sirtuins (SIRT1–7) are members of Class III HDACs, and the changes in SIRT activity or expression are closely associated with the pathophysiological changes and disease progression of acute and chronic kidney diseases, including AKI, DKD, PKD, and HTN (Perico et al., 2024).

This review aims to illustrate the roles and potential mechanisms of acetylation and deacetylation in the context of kidney physiology and pathophysiology and relative therapeutic targets. We also introduce the protective effects and mechanisms of the associated agonists and inhibitors in various kidney diseases, which may provide valuable insights into novel treatment strategies for renal pathologies.

## 2 Kidney disease

AKI is a syndrome characterized by a decline in renal function and high incidence, and the associated mortality and medical costs make AKI a global concern and a core research topic in epigenetics in the field of kidney disease (Zhuang et al., 2022; Nørgård and Svenningsen, 2023). The primary causes of AKI include infections, hypovolemic shock, and associations with sepsis, medications or invasive procedures, and the long-term complications of AKI include CKD, renal failure, and cardiovascular diseases, with an increased risk of mortality (Kellum et al., 2021; Ostermann et al., 2025). The core pathological features of AKI include renal tubular epithelial cell (RTEC) injury, inflammatory responses, and apoptosis (Lu J. et al., 2024; Ostermann et al., 2025). RTECs are particularly sensitive to various injury factors (obstruction, ischemia, hypoxia, oxidative stress, and metabolic disorders) due to their mitochondrial abundance and reliance on oxidative phosphorylation, which in turn leads to renal dysfunction (Li Z. L. et al., 2024). The surviving RTECs can promote the complete recovery of the kidneys through adaptive repair mechanisms after AKI. However, if the degree of injury is severe or the duration is long, it may trigger abnormal pathological reactions in RTECs, leading to dysregulation of the repair process and changes in cell phenotypes, thereby promoting the formation of renal fibrosis. This significantly increases the progression to CKD or end-stage renal disease (ESRD) (Xie et al., 2022; Li Z. L. et al., 2024).

**FIGURE 1:**
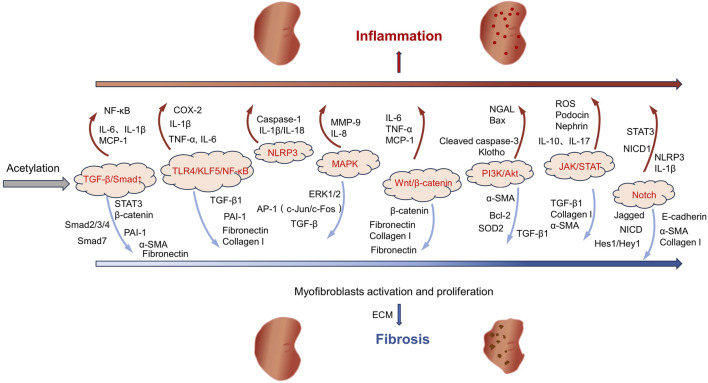
The role of acetylation in kidney inflammation and fibrosis.

The pathological process of AKI is often accompanied by systemic and local inflammatory responses, characterized by the overexpression of pro-inflammatory cytokines including tumor necrosis factor-alpha (TNF-α), interleukin-6 (IL-6), and interleukin-1 beta (IL-1β). The intensification of the inflammatory response is closely related to the activation of the Toll-like receptor 4 (TLR4)/Krüppel-like factor 5 (KLF5)/nuclear factor-kappa B (NF-κB) signaling pathway (Figure 1). This pathway not only promotes the activation of the NOD-like receptor family and pyrin domain-containing protein 3 (NLRP3) inflammasome but also further stimulates the secretion of inflammatory factors including IL-6, IL-1β, and TNF-α to aggravate tubulointerstitial inflammation and promote pyroptosis, ultimately exacerbating renal injury (Lu J. et al., 2024; Huang X. R. et al., 2025).

In the pathological process of AKI, apoptosis is mainly regulated in a coordinated manner through the mitochondrial pathway (endogenous apoptotic pathway), the death receptor pathway (exogenous apoptotic pathway), and the endoplasmic reticulum stress (ERS) pathway (Wang et al., 2022b; Jiang et al., 2024; Pei et al., 2024). The mitochondrial pathway can be triggered by hypoxia, ROS, and DNA damage, mainly leading to apoptosis by disrupting the balance of Bcl-2 family proteins. In this pathway, the transcription of p53 activates the expression of pro-apoptotic proteins Bax/Bak and inhibits the expression of anti-apoptotic proteins Bcl-2/Bcl-xL, resulting in increased mitochondrial membrane permeability and cytochrome c release, which in turn activates the caspase-9/3 cascade reaction and ultimately causes apoptosis (Aranda-Rivera et al., 2021; Wang et al., 2022b). In addition, the reduction in mitochondrial autophagy mediated by PINK1/Parkin leads to continuous ROS production and promotes apoptosis (Li T. et al., 2024; Zhang et al., 2025). The death receptor pathway directly activates caspase-8 by forming the Fas/tumor necrosis factor receptor 1 (TNFR1)-death inducing signaling complex, thereby activating caspase-3 or promotes apoptosis by amplifying the mitochondrial pathway (Li H. et al., 2021; Hu L. et al., 2025). ERS is a condition where the homeostasis of the endoplasmic reticulum is disrupted and is closely related to kidney diseases (Jiang et al., 2022). ERS is an intrinsic protective mechanism against external stressors. It occurs when the ER is unable to fully handle the accumulated misfolded proteins. Excessive ERS will cause cell death (Cheng et al., 2024). Furthermore, this process promotes apoptosis by activating the PERK/ATF4/CHOP signaling pathway, downregulating the expression of Bcl-2, and triggering the activation of caspase-12 (in mice) or caspase-4 (in humans) (Deng et al., 2023). Notably, these three pathways do not act alone but jointly promote RTEC apoptosis through crosstalk (such as the signal crossover between the mitochondria and death receptor pathways mediated by truncated Bid) and synergistic amplification effects, constituting the core regulatory network of AKI cell apoptosis.

Following kidney injury, a series of pathological processes occur, including mitochondrial dysfunction, oxidative stress, inflammation, fibrosis, and apoptosis, which may lead to CKD and ESRD (Fu et al., 2022). In the early stage of CKD, patients usually have no obvious symptoms. As the disease progresses, clinical manifestations such as decreased renal function and proteinuria will gradually appear. In the advanced stage, kidney function is often severely impaired, and eventually dialysis or kidney transplantation is needed to sustain life (Chen D. et al., 2024). DKD, hypertensive nephropathy (HTN), and polycystic kidney disease (PKD) are important factors leading to the development of CKD (Tang et al., 2020; Kalantar-Zadeh et al., 2021). Among these conditions, DKD is one of the primary reasons for the rapid increase in the incidence and mortality rates of CKD and its complications. DKD represents a significant complication in both type 1 and type 2 diabetes patients. It is the primary cause of CKD development and the leading cause of ESRD (Chen D. Q. et al., 2022; Liu et al., 2024a). Notably, DKD poses a heavy disease burden globally, significantly elevating the patients’ risk of kidney failure and cardiovascular events. This nephropathy originates from diabetes-induced dysregulation of glucose metabolism, which in turn triggers a series of metabolic abnormalities, hemodynamic changes, inflammatory responses, and fibrotic processes that ultimately drive disease progression (Tuttle et al., 2022). HTN is generally considered a consequence of chronic hypertension and is the second leading cause of ESRD. The pathogenesis of HTN is related to the persistent hypertension damaging renal tubular cells, which in turn leads to tubulointerstitial fibrosis (Wu J. C. et al., 2023). The clinical manifestations of the disease include nocturia, proteinuria, and a decrease in the glomerular filtration rate (GFR). Most patients develop mild-to-moderate hypertensive nephrosclerosis. However, those with uncontrolled chronic hypertension or pre-existing kidney disease are more likely to progress to CKD and eventually end up with ESRD (Wang L. et al., 2024). PKD, a rare renal disease that has a significant impact on patient care worldwide, encompasses a class of disorders presenting with bilateral cyst formation in the kidneys (Bergmann et al., 2018). This disease is caused by mutations in multiple genes, the most common of which are Pkd1, Pkd2, and PKHD1. The etiology of PKD may be genetic or nongenetic in origin, with inherited autosomal dominant polycystic kidney disease (ADPKD) affecting approximately 10% of dialysis patients and causing ESRD (Subhash et al., 2024; Boletta and Caplan, 2025). The primary manifestations of ADPKD are the formation of cysts within the kidneys and a gradual decline in renal function. Over time, these cysts continue to expand, potentially leading to a significant enlargement of both kidneys over the course of several years to several decades. During this process, approximately half of the ADPKD patients will progress to the stage of renal failure (Ramalingam and Patel, 2022; Chebib et al., 2024). The progression of CKD is related to intricate molecular and signaling pathways, and the key pathological mechanisms encompass fibrosis, apoptosis/senescence, and metabolic dysregulation (Abbad et al., 2025). The progression of fibrosis primarily involves the regulation of multiple signaling pathways, including TGF-β/Smad, MAPK, Wnt/β-catenin, PI3K/Akt, JAK/STAT, and Notch (Zhang et al., 2021a; Ansari et al., 2025) (Figure 1). Among these pathways, TGF-β/Smad, as the core regulatory axis, can phosphorylate Smad2/3 and form a complex with Smad4 to promote the transcription of fibrotic genes (including collagen I/III and fibronectin). Meanwhile, TGF-β downregulates Smad7 expression, relieves the negative regulation of Smad2/3, and amplifies the fibrosis signal (Huang et al., 2023; Soomro et al., 2023; Hong et al., 2025). The common feature of these pathways is that they can all be activated by inflammation, oxidative stress or mechanical injury and ultimately promote the activation of myofibroblasts, excessive deposition of extracellular matrix (ECM), and inhibit its degradation (Figure 1).

In the progression of CKD, apoptosis and aging jointly promote the deterioration of renal function. Apoptosis mainly works in synergy through the mitochondrial pathway (the upregulation of Bax/Bak and downregulation of Bcl-2 expressions leading to cytochrome C release), the death receptor pathway (the activation of caspase-8 mediated by Fas/FasL), and the ERS pathway (the inhibition of anti-apoptotic protein expression by the PKR-like endoplasmic reticulum kinase-C/EBP homologous protein (PERK-CHOP) pathway (Wang et al., 2022b; Jiang et al., 2024; Pei et al., 2024)). Senescent cells promote inflammation and fibrosis by activating the p16/p21-Rb pathway and secreting senescence-associated secretory phenotype (SASP) factors, including IL-1, IL-6, IL-8, TGF-β, plasminogen activator inhibitor (PAI), and insulin-like growth factor-1(IGF-1) (Lin et al., 2022; Tan et al., 2022; Zhao et al., 2022). It is worth noting that the DNA damage response caused by telomere shortening will further accelerate the aging process, forming a vicious cycle of “senescence-apoptosis-inflammation,” which jointly leads to loss of renal parenchymal cells and tissue repair disorders (Levstek and Trebušak Podkrajšek, 2023).

The metabolic disorders are mainly manifested as an energy imbalance and abnormal glucose and lipid metabolism. Disorders of energy metabolism are manifested as decreased adenosine monophosphate-activated protein kinase (AMPK) activity and excessive activation of mechanistic target of rapamycin (mTOR), which lead to the downregulation of key energy metabolism factors peroxisome proliferator-activated receptor alpha/proliferator-activated receptor gamma coactivator 1-alpha (PPAR-α/PGC-1α), causing mitochondrial dysfunction and lipid accumulation (Zhou S. et al., 2024). Lipid metabolism disorders are mainly manifested as the downregulation of PPAR expression and the increased activity of sterol regulatory element-binding protein 1c (SREBP-1c). This imbalance leads to the inhibition of the fatty acid oxidation pathway and the abnormal activation of the lipid synthesis pathway, thereby causing lipid deposition in the kidneys and intensification of inflammation (Li G. et al., 2021; Wang et al., 2022c; Yang G. et al., 2025). Glucose metabolism disorders are mainly driven by insulin resistance and accumulation of advanced glycosylation end products (AGEs), which lead to the occurrence of hyperglycemia and renal fibrosis by downregulating glucose transporter type 4 (GLUT4) expression and upregulating the receptor for advanced glycation end-products (RAGE)/NF-κB pathway. This pathological process is closely related to the regulation of the key signaling pathway PI3K/Akt/AGEs (Cao et al., 2025; Hou et al., 2025; Uribarri and Tuttle, 2025; Yang Z. et al., 2025). These metabolic disorders are intertwined, exacerbating inflammation and fibrosis, accelerating CKD progression.

CKD progresses slowly, regardless of the underlying cause, ultimately leading to irreversible loss of renal units, ESRD, and even potentially premature death. The deterioration of CKD is influenced by multiple factors, such as a reduction in parenchymal cells, persistent chronic inflammation, tissue fibrosis, and a decrease in the kidney’s ability to self-repair. Current treatment methods are limited in their effectiveness and can only slow down disease progression, which highlights the urgency and importance of developing new treatment modalities to prevent or reverse the progression of CKD (Ruiz-Ortega et al., 2020).

## 3 Acetylation and deacetylation in kidney disease

Acetylation, as one of the most extensively studied topics in PTM, primarily occurs on lysine residues and is categorized into two main types: histone acetylation and non-histone acetylation. Currently, the known types of acetylation include Nα-acetylation, Nε-acetylation, and O-acetylation. Nα-acetylation involves the addition of an acetyl group to the α-amino group at the N-terminus of the protein, a process that is irreversible. Nε-acetylation refers to the introduction of an acetyl group to the ε-amino group of lysine residues, a process that is reversible. O-acetylation involves the addition of an acetyl group to the hydroxyl group of tyrosine, serine or threonine. Among protein acetylation modifications, the acetylation of lysine residues is the most common form (Xia et al., 2020; Wang and Ma, 2025a).

**FIGURE 2:**
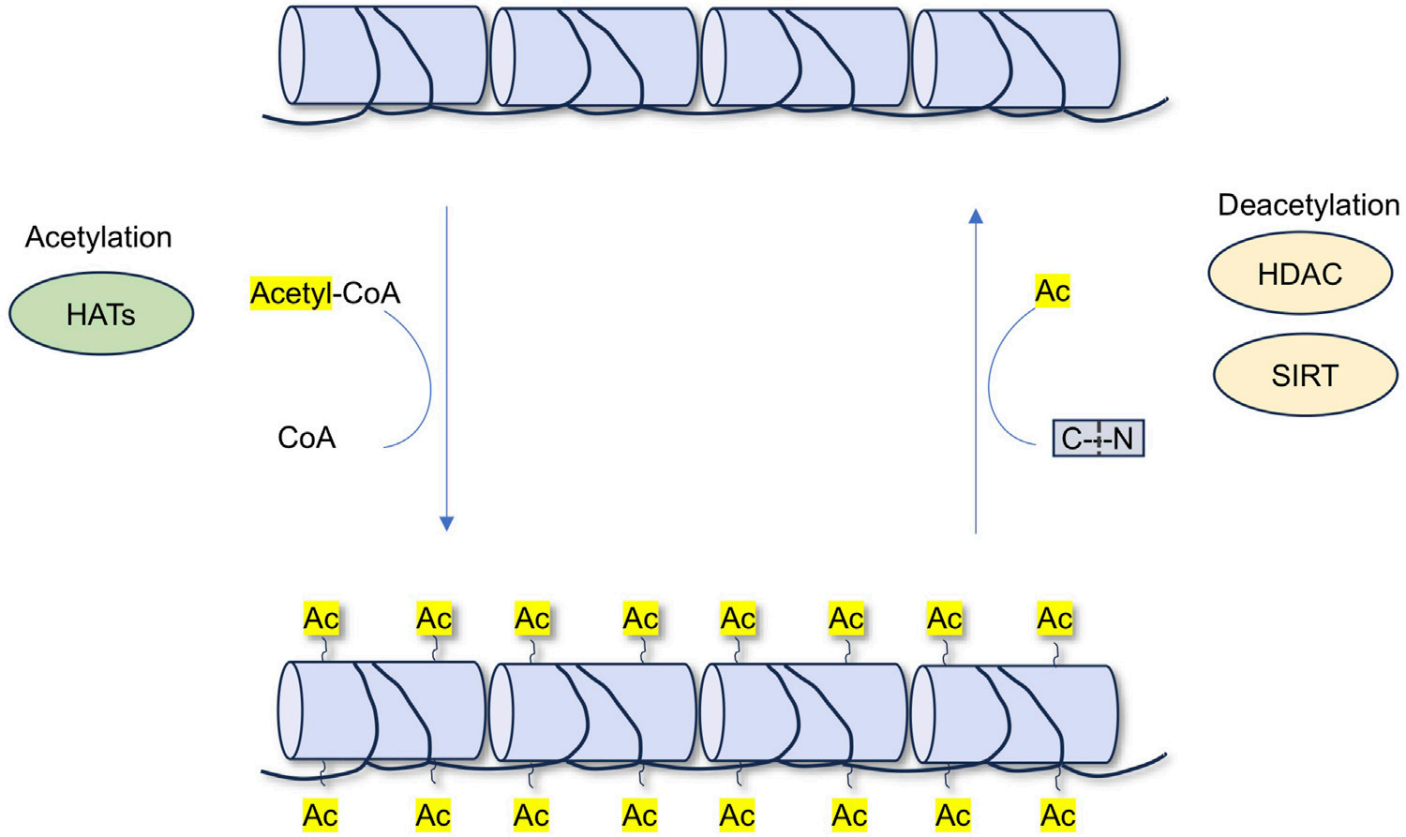
The process of acetylation and deacetylation.

HATs and HDACs jointly regulate acetylation, thereby influencing the physiological homeostasis of the kidneys. In recent years, HDACs have become a focus of research for therapeutic drugs targeting many kidney diseases (Shvedunova and Akhtar, 2022; Li X. Y. et al., 2023). HATs can introduce acetyl functional groups from acetyl donors (acetyl-CoA) into amino acid residues (including lysine), forming acetyl ester bonds and neutralizing their positive charges, leading to the relaxation of the nucleosome structure. This facilitates the specific binding of various transcription factors to DNA-binding sites and activates gene transcription. This process is dynamic and reversible (Dang and Wei, 2022; Wang and Ma, 2025a). Unlike HATs, which add acetyl groups to lysine residues and promote gene transcription, HDACs remove acetyl groups from proteins and induce transcriptional repression of genes (Figure 2).

Acetylation can modify the function of proteins by influencing their stability, activity, localization, and interactions with other proteins (Dang and Wei, 2022). Acetylation remolds protein structure and functions to play a central role in multiple biological processes, including cell metabolism, proliferation, differentiation, and apoptosis (Jiang et al., 2023). Deacetylation is the opposite process of acetylation and is mediated by deacetylases (Chen et al., 2020). Together, protein acetylation and deacetylation play important roles in renal disease.

### 3.1 Acetylation and deacetylation in acute kidney injury

#### 3.1.1 HDACs in AKI

HDACs play an important role in acetylation by silencing the gene expression, regulating histone lysine deacetylation or forming co-repressor complexes with transcription factors. However, HDACs can also interact with non-histones and deacetylate them, thereby regulating cellular functions. HDACs are classified into four classes: class I (HDAC1, HDAC2, HDAC3, and HDAC8), class II (HDAC4, HDAC5, HDAC6, HDAC7, HDAC9, and HDAC10), and class IV HDAC (only the HDAC11) are Zn^+^-dependent enzymes, and class III are NAD^+^-dependent deacetylases (Table 1).

**TABLE 1:** The classification of HDACs and their role in renal disease.

Classification	Members		Cofactor dependency	Location	Renal diseases			References
					Type	Model	↑/↓	
Class I	HDAC1		Zn^2+^	Nucleus	AKI	Cisplatin-AKI mice/TKPTs	↑	Ranganathan et al. (2016)
					DKD	STZ-DKD mice/HG-GMCs		Hao et al. (2021)
	HDAC2				AKI	RIRI-mouse	↑	Aufhauser et al. (2021)
						AKI patients/LPS-HK-2 cells		Xie et al. (2023)
					DKD	db/db mice/TGF-β-HK-2 cells	↑	Zheng et al. (2022)
						db/db mice/HG- NRK-52E cells		Du et al. (2020)
	HDAC3				AKI-CKD	AA and FA-mice/AA-HK-2 cells	↑	Zhang et al. (2023a)
					DKD	db/db mice/HG- SV40-MES-13	↑	Zhang et al. (2024b)
	HDAC8				AKI	Cisplatin-mice/cisplatin-RTECs	↑	Wang et al. (2024c)
Class II	Class IIa	HDAC4		Nucleus and Cytosol	AKI	Sepsis-mice/LPS- CD4^+^T	↑	Liu and Chen (2024c)
						Contrast agent (ioversol)-mice/HK-2cells		Zuo et al. (2024)
					DKD	DKD patients/HG-podocytes	↑	Shi et al. (2020)
						DKD patients/HG-HK-2 cells		Huang et al. (2024)
		HDAC5			AKI	Oxalate nephropathy-mice/LPS-macrophages	↑	Sharma et al. (2022)
					DKD	UUO-mice/HG-HK-2 cells	↑	Xu et al. (2021b)
		HDAC9			DKD	DKD patients	↑	Fan et al. (2020a)
	Class IIb	HDAC6			AKI	Ischemia/cisplatin-mice	↑	Shi et al. (2022), Zhang et al. (2023b)
						Rhabdomyolysis mice/cisplatin-HK-2 cells		Shi et al. (2017), Tang et al. (2018)
					DKD	Type 2 diabetic mice/db/db mice/AGE-podocytes	↑	Liang et al. (2020)
						DKD patients/STZ-mice/TGF-β-HK-2 cells		Hou et al. (2022)
					ADPKD	Pkd1 mutation-MEK epithelial cells	↑	Liu et al. (2012)
						Pkd1 mutation mice		Yao et al. (2021)
Class IV	HDAC11			Cytoplasm	AKI	I/R-mice/LPS-macrophages	↓	Núñez-Álvarez and Suelves (2022)

##### 3.1.1.1 HDAC1

The expression of HDAC1 is significantly upregulated in the cisplatin-induced AKI model and the immortalized mouse proximal tubule cells (TKPTs) treated with cisplatin, accompanied by renal tubular necrosis, tubular cell formation, and marked epithelial cell apoptosis. The upregulation of HDAC1 expression leads to the silencing of microglia/macrophage WAP domain protein (AMWAP), which further results in epithelial cell apoptosis, kidney damage, and inflammatory responses. However, the administration of a HDAC1-specific inhibitor can increase the level of histone acetylation in the AMWAP promoter region, enhance the transcriptional activity of the AMWAP gene, promote the expression of AMWAP, and effectively protect the kidney from cisplatin-induced AKI damage (Ranganathan et al., 2016). HDAC1 plays a crucial role in the protective effects of 7-hydroxycoumarin against colistin-induced kidney injury. Specifically, colistin significantly promotes the upregulation of HDAC1 expression and leads to the deacetylation of histone 3 lysine 27 (H3K27) in the Nrf2 (the master regulator of anti-oxidant response) promoter region to inhibit the activity of the Nrf2 signaling pathway. In addition, renal tubular expansion, epithelial cell detachment, and necrosis are observed in a colistin-induced renal injury mice model. However, the treatment with 7-hydroxycoumarin reduces the expression level of HDAC1 and activates the Nrf2 signaling pathway, significantly enhancing the kidney’s antioxidant capacity and thus exerting a protective effect on the kidney (Wang et al., 2020). These results indicate that the inhibition of HDAC1 may provide a protective effect against kidney injury induced by colistin.

##### 3.1.1.2 HDAC2

The tamoxifene-induced specific knockout of HDAC2 can effectively protect against renal injury and alleviate renal fibrosis caused by renal ischemia reperfusion injury (RIRI). HDAC1 and HDAC2 contribute to several nuclear multimeric coregulatory complexes, including CoREST. The absence of HDAC2 makes the CoREST-HDAC1 complex more stable to exert the renal protective effect. Additionally, HDAC2 deficiency also leads to decreased expression of Edn1 and Ednra (endothelin receptor type A) to alleviate renal injury. Notably, the pharmacological inhibition of HDAC2 does not improve renal function or attenuate renal fibrosis. Therefore, the renal protective effect brought about by the deficiency of HDAC2 may not be related to the HDAC2 deacetylase catalytic activity (Aufhauser et al., 2021). The HDAC2 deficiency alleviates IRI by reducing endothelin expression through interaction with Dot1l. In the kidney, the endothelin is produced by endothelial cells and podocytes in the thylakoid and glomerular basement membranes (GBM). The endothelin system regulates glomerular function and contributes to the exacerbation of IRI through the TNF-α-mediated pathway (Aufhauser et al., 2021; Rakotoarison et al., 2024). The expression of HDAC2 is increased in AKI patients and lipopolysaccharide (LPS)-treated HK-2 cells. The regulatory role of extracellular vesicles (EVs) derived from bone marrow mesenchymal stem cells (BMSCs) in inflammation and pyroptosis during AKI is closely related to HDAC2. Specifically, BMSC-EV-encapsulated microRNA-223-3p (miR223-3p) mitigates AKI-induced inflammation and pyroptosis by inhibiting HDAC2 and increasing H3 acetylation to promote sucrose non-fermenting-1 (SNF1)-related kinase (SNRK) transcription (Xie et al., 2023).

##### 3.1.1.3 HDAC3

HDAC3 participates in the progression of AKI to CKD that includes an adverse adaptive response and multiple complex mechanisms. These mechanisms include endothelial cell damage, inflammatory response exacerbation, fibrosis progression, and other pathways that promote disease occurrence and development (Wang and Zhang, 2022e). Ferroptosis is a novel form of non-apoptotic cell death with a significant role in the progression of AKI to CKD (Guo R. et al., 2023). Ferroptosis is primarily characterized by the iron-dependent accumulation of lipid peroxides and mitochondrial membrane damage. Various factors can induce ferroptosis, including impaired amino acid and lipid metabolism, iron handling, mitochondrial activity or redox reactions. In this process, the dysregulation of glutathione (GSH)/glutathione peroxidase 4 (GPX4) signaling plays a crucial role. GPX4 converts lipid hydroperoxides (L-OOH) into lipid alcohols (L-OH), effectively blocking the progression of ferroptosis (Martin-Sanchez et al., 2020; Zhang L. et al., 2023; Park et al., 2025). In a mouse model of AKI-CKD transformation induced by nephrotoxic substances aristolochic acid (AA) and folic acid (FA) and the HK-2 cells treated with AA, the increased expression of HDAC3 leads to the downregulation of GPX4 mRNA, thereby promoting the progression from AKI to CKD. Notably, the knockout of the HDAC3 gene or the use of HDAC3 inhibitors can effectively alleviate the inhibition of GPX4, ferroptosis, and renal function damage associated with fibrosis (Zhang L. et al., 2023).

##### 3.1.1.4 HDAC4

The expression of HDAC4 is upregulated in the CLP-induced sepsis AKI (SAKI) mice model and the CD4^+^T cells treated with LPS. Human umbilical cord-derived mesenchymal stem cell (HUCMSC)-exosomes (EXOs) deliver miR-375 to CD4^+^T cells and inhibit HDAC4 expression to increase the beclin-1 and LC3-II/LC3-I expression, promoting autophagy and inhibiting T-cell apoptosis and mitigating pathological changes, including the edema of RTECs, necrosis of renal tubules, and severe congestion induced by SAKI (Liu and Chen, 2024c). In addition, the expression of HDAC4 is also significantly upregulated, accompanied by a series of pathological damages including tubular lumen dilation, the diminished tubular epithelial cell abundance, and inflammatory cell infiltration in ioversol-induced AKI mice model and HK-2 cells exposed to ioversol. Berberine can reverse the increased expression of HDAC4 and ameliorates contrast-induced pathological injury. Mechanically, berberine reduces the increased acetylation level of FoxO3a (Ac-FoxO3a) and phosphorylation of FoxO3a (p-FoxO3a), inhibits the Bcl-2-associated X protein (Bax) expression, and restores Bcl-2 expression. Berberine increases the formation of autophagosomes and autolysomes, reduces the expression level of LC3II/LC3I and beclin-1 protein, and increases the P62 protein expression to enhance cellular autophagy (Zuo et al., 2024). Therefore, targeting the HDAC4-FoxO3a axis may be a therapeutic strategy for contrast agent-induced AKI.

##### 3.1.1.5 HDAC5

The increased expression of HDAC5 and NLR family pyrin domain-containing 3 (NALP3) and the decreased expression of kruppel-like factor 2 (KLF2) are observed in the sodium oxalate-induced acute oxalate nephropathy mouse model and murine macrophages treated with LPS/sodium oxalate. The downregulation of HDAC5 expression with RNA interference can improve renal tubular injury by upregulating the expression of KLF2 and inhibiting the expression of NALP3 in a sodium oxalate-induced acute oxalate nephropathy mouse model. Based on this mechanism, HDAC5 downregulation protects renal function by inhibiting neutrophil accumulation, decreasing the expression of AKI markers kidney injury molecule-1 (KIM-1) and neutrophil gelatinase-associated lipocalin (NGAL), and downregulating pro-caspase 1, active caspase 1, and IL-1β expression to mitigate tubular cell damage (Sharma et al., 2022).

##### 3.1.1.6 HDAC6

HDAC6 is found exclusively in the cytoplasm and deacetylates cytoplasmic proteins, including α-tubulin. HDAC6 is involved in the pathology of renal diseases by exerting its deacetylation-dependent/non-dependent effects on a wide range of target molecules. HDAC6 is significantly activated in ischemia and cisplatin-induced AKI models. Mechanically, HDAC6 exacerbates AKI induced by ischemia and cisplatin through the inhibition of autophagy and α-tubulin acetylation (Shi et al., 2022; Zhang Q. Q. et al., 2023). In the glycerol-induced rhabdomyolysis mouse AKI model and HK-2 cells treated with cisplatin, renal tubule dilation, swelling, necrosis, and lumen congestion are observed, accompanied by the increased HDAC6 and NGAL expression. The inhibition of HDAC6 reverses these changes, promotes autophagy, and reduces oxidative stress, thereby alleviating kidney damage. Mechanically, the inhibition of HDAC6 enhances the acetylation of histone 3 and reduces the NGAL and caspase-3 expression in mice kidney to mitigate the effects of renal injury and apoptosis. Furthermore, inhibiting HDAC6 significantly reverses the increase in glycerol-induced nuclear factor kappa B (NF-κB) phosphorylation level and alleviates the inflammatory response (Shi et al., 2017; Tang et al., 2018).

##### 3.1.1.7 HDAC8

The increased expression of HDAC8 is observed in a cisplatin-induced AKI mice model and RTECs exposed to cisplatin, accompanied with significant tubular injury and renal cell apoptosis. Notably, the pharmacological and genetic inhibition of HDAC8 alleviates cisplatin-induced AKI by reducing DNA damage and promoting its repair. Specifically, inhibiting HDAC8 can effectively reduce the cleavage of caspase-3 and polyadenosine diphosphate ribose polymerase 1 (PARP1), decrease the expression of Bax, and maintain the level of Bcl-2 in injured kidneys to reduce cell apoptosis. Furthermore, the pharmacological inhibition of HDAC8 can effectively suppress p53 and p21 expressions and the phosphorylation of the cyclin-dependent kinase 2 (CDK2) induced by cisplatin to reduce the expression of pro-apoptotic genes, thus alleviating apoptosis and cell cycle arrest (Wang et al., 2024c).

##### 3.1.1.8 HDAC11

HDAC11, the latest member of the HDAC family and the only Class IV HDAC, is a multifaceted enzyme with very potent long-chain fatty acylase deacetylase activity (Liu S. S. et al., 2020; Núñez-Álvarez and Suelves, 2022). It is highly expressed in the brain, heart, kidneys, and several other organs, and is located in the mitochondria, cytoplasm, and nucleus (Chen H. et al., 2022). The expression of HDAC11 is decreased but the PAI-1 (a key pathophysiological regulatory factor, involved in processes including inflammation, fibrosis and apoptosis) expression is increased in the ischemia/reperfusion (I/R)-induced AKI male mice model and LPS-treated macrophages. Notably, the downregulation of HDAC11 expression and the upregulation of PAI-1 expression are reversed in the male mice undergoing orchiectomy treated with I/R. Therefore, the decreased expression of HDAC11 and the increased expression of PAI-1 induced by I/R are testosterone-dependent. The reduced expression of HDAC11 leads to the increase in H3ac levels in the PAI-1 promoter and enhances PAI-1 expression, which aggravates I/R-induced inflammatory responses and kidney injury. This entire process is androgen-dependent, indicating gender differences in renal I/R injury (Núñez-Álvarez and Suelves, 2022). Based on the negative regulatory effects of HDAC11 on PAI-1, the use of HDAC11 agonists is very likely to protect I/R-induced renal injury by upregulating the expression of HDAC11 and inhibiting the expression of PAI-1, thus becoming a potential therapeutic strategy for I/R-induced AKI.

#### 3.1.2 SIRTs in AKI

SIRTs are important regulators of cell function and organismal health belonging to an evolutionarily conserved family, classified by the type of the enzymatic reaction as deacetylases, deacylases, and ADP-ribosyltransferases (Juszczak et al., 2024). SIRTs are distributed in three main locations, SIRT1 and SIRT2 in the nucleus and cytoplasm; SIRT3, SIRT4, and SIRT5 in the mitochondria; and SIRT6 and SIRT7 in the nucleus (Table 2). The progression of kidney disease is closely related to the abnormal expression of the SIRT proteins. Most SIRTs can effectively promote the recovery of renal function and delay the progression of kidney disease by regulating the expression and activity of proteins (Jin et al., 2024). However, the expressions of SIRT2 and SIRT7 are significantly upregulated in AKI, thereby exacerbating kidney damage. Therefore, the overexpression or increased activity of SIRT2 and SIRT7 is detrimental to AKI (Jung et al., 2020; Chen G. et al., 2022; Sánchez-Navarro et al., 2022; Yu et al., 2025).

**TABLE 2:** The classification of SIRTs and their role in renal disease.

Classification	Members	Cofactor dependency	Location	Renal diseases			References
				Type	Model	↑/↓	
Class III	SIRT1	NAD^+^	Nucleus and cytoplasm	AKI	Sepsis mice/LPS-HK-2 cells	↓	Deng et al. (2021)
					LPS mice		He et al. (2022)
				DKD	db/db mice/HG-HK-2 cells		Du et al. (2021)
					STZ mice/HG-HK-2 cells	↓	Sun et al. (2021)
					HFD mice/HG- SV40 MES13		Wang et al. (2023a)
					Db/db mice/HG-MPCs		Su et al. (2022)
					RIRI mice		Feng et al. (2023)
				HTN	SHRs/Ang II-NRK-52E cells	↓	Cai et al. (2021), Zhao et al. (2024)
					H_2_O_2_-induced NRK-52E cells		Ko et al. (2023)
				PKD	Pkd1 mutant-mice	↑	Zhou et al. (2013)
	SIRT2			AKI	Cisplatin mice	↑	Jung et al. (2020)
					Sepsis mice/LPS-HK-2 cells	↑	Yu et al. (2025)
				DKD	HG podocytes MPC5	↑	Liu et al. (2022a)
					HFD/STZ mice/HG/PA-HK-2 cells	↑	Chen et al. (2025)
					DKD patients/UUO mice	↓	Yang et al. (2023)
				HTN	Ang-II mice	↑	Dikalov and Kirabo (2023), Zhang et al. (2023d)
	SIRT3		Mitochondria	AKI	Lovesol-mice/Lovesol-HK-2 cells	↑	Zhang et al. (2022b)
					APAP-mice/APAP-HK-2 cells	↓	Wei et al. (2022b)
				CKD	Sepsis mice/LPS-RTECs	↓	Fan et al. (2024)
				HTN	HSD mice/Ang II-HK-2 cells	↓	Wang et al. (2023e), Zhang et al. (2023d)
	SIRT4			AKI	I/R mice/OGD/R-HK-2 cells	↓	Liu et al. (2025)
				DKD	DKD patients/HG podocytes	↓	Xu et al. (2021a)
					HG/HFD db/db mice		Liang et al. (2023)
	SIRT5			AKI	Cisplatin-HK-2 cells	↓	Li et al. (2019)
					IRI mice/OND-hPTECs	↑	Haschler et al. (2021)
	SIRT6		Nucleus	AKI	Sepsis mice	↓	Gao et al. (2021)
				DKD	HG db/db mice/HG podocytes	↓	Du et al. (2024), Zhang et al. (2024c), Ma et al. (2025)
					STZ mice		Liu et al. (2017), Elrashidy et al. (2024)
				HTN	Deoxycorticosterone acetate/salt/Ang-II-hypertensive mouse	↓	Guo et al. (2019)
					Ang-podocytes		Yang et al. (2020), Fan et al. (2021)
	SIRT7			AKI	IRI mice	↑	Sánchez-Navarro et al. (2022)
					Cisplatin mice/HK-2 cells		Chen et al. (2022b)
					I/R mice	↓	Wang et al. (2023d)
				DKD	HG-podocytes	↓	Qi et al. (2022a)
					HG HGECs/STZ mice		Wu et al. (2024a)
					DKD patients/HG-HGECs		Lu et al. (2024b)
				HTN	Ang-II mice	↓	Li et al. (2022c)

##### 3.1.2.1 SIRT1

The SIRT1 expression is downregulated in CLP-induced SAKI and HK-2 cells stimulated by LPS, accompanied by the renal dilation, with epithelial cell flattening and shedding. The activation of SIRT1 promotes autophagy by deacetylating the beclin 1 at the K430 and K437 sites to attenuate SAKI (Deng et al., 2021). The expression of SIRT1 is decreased, and pathological changes including renal tubular injury and mitochondrial and autophagic dysfunction are observed in the IRI-induced AKI mice model. The protective effect of SIRT1 is closely related to cytosolic NAD^+^ (Morevati et al., 2021). The renoprotection of NAD^+^ is SIRT1-dependent, and NAD^+^ can attenuate LPS-induced AKI by modulating the glycogen synthase kinase-3β (GSK-3β)/Nrf2 pathway. The inhibition of SIRT1 reduces the protective effect of NAD^+^, leading to an increase in GSK-3β activity and attenuation of Nrf2 nuclear accumulation, further exacerbating AKI. Therefore, the NAD^+^/SIRT1/GSK-3β/Nrf2 axis is an important mechanism for AKI prevention and may be a potential therapeutic target for AKI treatment (He et al., 2022).

##### 3.1.2.2 SIRT2

The expression of mitogen-activated protein kinase phosphatase-1 (MKP-1) is reduced, and the expression of SIRT2 is increased in the cisplatin-induced AKI mice model. The knockout of SIRT2 can reverse the cisplatin-induced downregulation of MKP-1, inhibiting the phosphorylation of p38 and c-Jun N-terminal kinase (JNK) in renal and tubular epithelial cells, alleviating cisplatin-induced tubular damage, apoptosis, and inflammation, thereby improving renal function (Jung et al., 2020).

The expression of SIRT2 is upregulated in the cecal ligation and puncture (CLP)-induced SAKI mouse model and in LPS-treated HK-2 cells, accompanied by significant tubular damage, characterized by the detachment of RTECs, tubular dilation, and epithelial cell flattening. During SAKI, the inhibition and knockout of SIRT2 enhances autophagy in RTECs and reduces the production of pro-inflammatory factors including IL-6, TNF-α, and IL-1β, thereby alleviating SAKI. Mechanically, SIRT2 interacts with forkhead box protein O1 (FoxO1); decreases the acetylation level of FoxO1 at K262, K265, and K274 sites; and inhibits its translocation from the nucleus to the cytoplasm, thereby inhibiting autophagy in RTECs and exacerbating SAKI (Yu et al., 2025).

##### 3.1.2.3 SIRT3

SIRT3 is a major mitochondrial deacetylase that regulates the acetylation of substrates that control metabolism and oxidative stress. In the kidney, renal tubular cells and podocytes are more susceptible to SIRT3 because of their high energy demand (Pezzotta et al., 2023). The SIRT3 expression is upregulated in loversol-induced AKI and HK-2 cells treated with loversol, accompanied by the lumen congestion and the interstitial edema of renal tubules. In the murine model where SIRT3 is selectively deleted, a rise in serum creatinine (SCr) and blood urea nitrogen (BUN) level is observed, but the activities of superoxide dismutase (MnSOD) and catalase are reduced. The expression of caspase-3 and the level of ROS are significantly increased, while the ratio of the anti-apoptotic protein Bcl-2 to the pro-apoptotic protein Bax is significantly decreased. SIRT3 potentially serves as a novel therapeutic target for the mitigation of kidney damage induced by contrast agents (Zhang Q. et al., 2022). Notoginsenoside Fc (Fc) is a novel protopanaxadiol-type (PPD-type) saponin isolated from ginseng leaves. Fc can effectively reduce the levels of SCr, BUN, and cystatin C in AKI mice to exert protective effects on the kidneys (Guo Z. et al., 2023). In the acetaminophen (APAP)-induced AKI model and HK-2 cells treated with APAP, the protein expressions of SIRT3 and SOD2 are reduced, while that of Ac-SOD2 is increased. Fc can restore the protein levels of SIRT3, SOD2, and Ac-SOD2; reduce oxidative stress; and alleviate tubular and mitochondrial damage in APAP-induced AKI (Wei M. et al., 2022). The decreased expression of SIRT3 and the increased expression of SCr, p-NGAL, and p-KIM-1 are observed in the CLP-induced SAKI mice model and the RTECs treated with LPS. The SIRT3 deficiency triggers a decline in renal function, a significant increase in inducible nitric oxide synthase (iNOS), and upregulation of the expressions of toll-like receptor 4 (TLR4), NF-κB, inhibitor of Kappa Bα (IκBα), inhibitor of kappa B kinase (IKKβ), and p65 proteins. However, N-acetylcysteine treatment significantly improves renal function and decreases the expression levels of TLR4, IκBα, IKKβ, and p65 proteins. Additionally, N-acetylcysteine increases the expression of Bcl-2 to inhibit apoptosis in KTEC cells by upregulating SIRT3 expression to exert protective effect against SAKI (Fan et al., 2024).

##### 3.1.2.4 SIRT4

The expression of SIRT4 is downregulated in the I/R-induced AKI mice model and the HK-2 cells treated with oxygen-glucose deprivation/reoxygenation (OGD/R), accompanied with autophagy reduction. Knocking out SET domain bifurcated histone lysine methyltransferase 1 (SETDB1) reduces the binding of SETDB1 to the SIRT4 promoter at the BS1 site, thereby upregulating SIRT4 expression to reduce the phosphatase and tensin homolog deleted on chromosome 10 (PTEN) expression and increasing the expression of autophagy markers (including LC3II/I and beclin 1) and p62 levels to promote autophagy, thereby alleviating I/R-induced renal injury. In addition, knocking out SETDB1 reduces the binding of chromobox protein homolog 3 (CBX3) to the SIRT4 promoter, thereby increasing the expression of SIRT4. This reduces the expression of PTEN, promoting autophagy and ultimately protecting rats from I/R damage (Liu et al., 2025).

##### 3.1.2.5 SIRT5

SIRT5 is a pivotal enzyme that promotes metabolic homeostasis, inhibiting mitochondrial fission and degradation processes, reducing the susceptibility of the mitochondria to oxidative stress-induced mitochondrial dysfunction. Since the repair and remodeling of mitochondrial function are key determinants in the progression of AKI, it may govern the transition from AKI to CKD (Haschler et al., 2021). In cisplatin-treated HK-2 cells, the expression of SIRT5 is decreased, accompanied by the decrease in mitochondrial metabolic activity and increase in apoptosis and oxidative stress. The upregulation of SIRT5 expression alleviates apoptosis and mitochondrial damage induced by cisplatin in HK-2 cells by modulating the Nrf2/HO-1 and Bcl-2 pathways. Mechanically, the SIRT5 overexpression suppresses caspase-3 cleavage and cytochrome c release. Additionally, SIRT5 upregulates the expressions of Nrf2, HO-1, and the anti-apoptotic protein Bcl-2, but it downregulates the pro-apoptotic protein Bax expression. These changes lead to increased mitochondrial membrane potential and reduced intracellular ROS generation to reduce oxidative stress. The inhibition of SIRT5 expression will have the opposite effect (Li et al., 2019). The expression of SIRT5 is increased in the proximal tubular epithelial cells (PTECs) of mice under IRI and in the human proximal tubular epithelial cells (hPTECs) exposed to oxygen/nutrient deprivation (OND). The absence of SIRT5 inhibits ATP production, results in the decrease in mitochondrial membrane potential, and leads to mitochondrial fragmentation in hPTECs. Additionally, the inhibition of SIRT5 exacerbates the mitochondrial bioenergetic function damage and swelling induced by OND and promotes mitochondrial autophagy reduction (Haschler et al., 2021). Currently, research studies on SIRT5 inhibiting the progression of AKI through the regulation of acetylation pathways are relatively limited, with a significant number of studies focusing on the impact on liver or lung diseases by adjusting phosphorylation or succinylation pathways. However, as previously mentioned, as a key regulator of mitochondrial function, SIRT5 is likely to have a potential interventional role in AKI caused by various factors.

##### 3.1.2.6 SIRT6

SIRT6 plays the role of an antioxidant in renal toxicity, IRI, obstructive nephropathy, and SAKI. Additionally, SIRT6 is important in the transition from AKI to CKD and renal repair through anti-inflammatory, anti-fibrotic, and mitochondrial quality control mechanisms (Yang et al., 2021). In the CLP-induced SAKI mouse model, the expression levels of SIRT6 and ubiquitin-specific peptidase 10 (USP10) in kidney tissue are decreased, accompanied by severe vacuolar degeneration, cell shedding, and inflammatory cell infiltration in the RTECs. The overexpression of USP10 significantly increases the expressions of SIRT6, Nrf2, and HO-1 in LPS-treated HK-2 cells, effectively reducing the shedding and local vacuolar degeneration of RTECs, the number of inflammatory cells, and improving renal function. Mechanistically, USP10 alleviates LPS-induced renal functional damage by activating the SIRT6-dependent Nrf2/HO-1 signaling pathway to reduce the levels of H_2_O_2_ and MDA, inhibiting the Bax and cleaved caspase-3 expressions and elevating the Bcl-2 expression. These changes indicate that oxidative stress, renal injury, and apoptosis induced by CLP are alleviated (Gao et al., 2021).

##### 3.1.2.7 SIRT7

The SIRT7 expression is increased in mouse kidney tissue after I/R treatment, accompanied by renal insufficiency, renal tubule damage, albuminuria, oxidative stress, and kidney inflammation. The knockout of SIRT7 protects mice from I/R-induced AKI through inhibiting the activation of NF-κB pathway and reducing the expression of pro-inflammatory factors IL-6, TNF-α, and monocyte chemoattractant protein-1 (MCP-1). The anti-inflammatory effect is associated with the decreased expression of p65 and the increased phosphorylation of p65. Therefore, the SIRT7 inhibitors may be an attractive therapeutic option for AKI induced by I/R (Sánchez-Navarro et al., 2022). The significant upregulation of p53 and SIRT7 and the downregulation of miR-142-5p are observed in the cisplatin-induced AKI mice model and the HK-2 cells treated with cisplatin, accompanied with significant renal tubular injury and apoptosis. Notably, the inhibition of p53 enhances miR-142-5p expression, further inhibiting the activation of the SIRT7 and NF-κB signaling pathway, preventing cisplatin-induced apoptosis to alleviate cisplatin-induced AKI (Chen G. et al., 2022). However, SIRT7 also has kidney-protective effects in I/R-induced AKI. The upregulation of miR-152-3p inhibits the SIRT7 expression, increasing the expression of exogenous apoptosis molecules including FoxO3a, Bcl-2-interacting mediator of cell death (Bim), and caspase-3, ultimately aggravating I/R-induced apoptosis and functional impairment of renal cells. However, the inhibition of miR-152-3p restores SIRT7 expression to reverse these processes (Wang et al., 2023d). Therefore, SIRT7 has a dual effect on AKI.

### 3.2 Acetylation and deacetylation in chronic kidney disease

#### 3.2.1 HDACs in chronic kidney disease

##### 3.2.1.1 HDAC1

**Diabetic kidney disease.** The expression of HDAC1 is increased, but the expression of miR-125a is decreased in a streptozotocin (STZ)-induced DKD mice model and high glucose (HG)-treated glomerular mesangial cells (GMCs). Notably, the exosome-derived mesenchymal stem cell carries miR-125a to mitigate the effects of DKD by inhibiting the HDAC1/endothelin-1 (ET-1) axis. Specifically, the miR-125a directly binds to HDAC1 and reduces the expression of ET-1, thereby alleviating the pathological symptoms including mesangial matrix expansion, capillary lumen shrinkage, inflammatory cell infiltration, and renal tubule injury in DKD rats (Hao et al., 2021).

**Renal fibrosis.** The expression of HDAC1 is upregulated, and the expression of dual-specificity phosphatase 1 (DUSP1) is downregulated in the unilateral ureteral obstruction (UUO)-induced renal fibrosis mouse model and HK-2 cells treated with TGF-β. The deficiency of DUSP1 exacerbates renal injury and inflammatory response and promotes nuclear translocation of small mothers against decapentaplegic homolog 3 (Smad3), leading to the aggravation of UUO-induced fibrosis. Interestingly, the DUSP1 expression is regulated by HDAC1. The inhibition of HDAC1 expression increases the expression of DUSP1, significantly reduces fibrosis markers, and hinders the phosphorylation of Smad3, thus alleviating UUO-induced renal tubules injury and renal fibrosis (Wang S. et al., 2025).

##### 3.2.1.2 HDAC2

**Diabetic kidney disease.** The accumulation of extracellular matrix (ECM) is one of the key pathological markers of DKD (Chen Y. et al., 2022). The regulatory network composed of HDAC2 and miR-205 affects the development of DKD by controlling the production of the ECM in RTECs. The decreased expression of miR-205 is observed in the HK-2 cells treated with TGF-β1 and db/db mouse model (a spontaneous model of DKD). Notably, the expression and activity of miR-205 are regulated by HDAC2. HDAC2 inhibits the activity of the miR-205 promoter by reducing the acetylation of H3K9 in the promoter region and downregulates miR-205 expression through the specific protein 1 (Sp1)-mediated pathway. In turn, miR-205 can directly target HDAC2 to inhibit its expression and regulate its own transcription by increasing the acetylation of H3K9 in the promoter region, forming a feedback regulatory loop. In summary, the HDAC2/SP1/miR-205 feedback loop plays a crucial role in the pathological mechanism of DKD (Zheng et al., 2022). The expression and activity of HDAC2 are increased in db/db mice and HG-treated NRK-52E cells. However, the sodium butyrate significantly reduces the expression and activity of HDAC2, mitigates cell apoptosis in db/db mice, and inhibits apoptosis and oxidative stress induced by HG in NRK-52E cells. At the molecular level, sodium butyrate inhibits the ROS production and LHH level, but increases SOD level in HG-treated NRK-52E cells to reduce oxidative stress and inhibit the expression and activity of HDAC2 (Du et al., 2020).

**Renal fibrosis.** The expressions of HDAC1, HDAC2, and the early growth response 1 (EGR1) are increased in doxycycline-induced podocyte-specific Tln1-knockout mice. Notably, the pharmacological inhibition of HDAC1 and HDAC2 mitigates the increased expression of EGR1 mediate by cAMP response element-binding protein (CREB). In addition, the inhibition of HDAC1 and HDAC2 alleviates glomerular sclerosis, interstitial fibrosis, and doxycycline-induced podocyte damage (Inoue et al., 2019).

##### 3.2.1.3 HDAC3

**Diabetic kidney disease.** The HDAC3 expression is upregulated in the db/db mice kidney and HG-treated SV40-MES-13 cells, accompanied with tubule dilation and glomerular hypertrophy. The inhibition of HDAC3 by astragaloside I effectively improves the renal function of db/db mice and alleviates the renal fibrosis induced by HG in SV40-MES-13 cells. The potential mechanism is related to that astragaloside I simultaneously inhibits the HDAC3 and TGF-β1 activity, regulating the Klotho/TGF-β1/Smad2/3 signaling pathway mediated by HDAC3. Notably, in the db/db- and the HG-treated SV40-MES-13 cells, the protective effect of astragaloside I on drug-induced renal fibrosis mediated by the Klotho/TGF-β1/Smad2/3 pathway can be offset by the ITSA-1 (a HDAC3 agonist) (Zhang X. et al., 2024).

**Renal fibrosis.** The expression of HDAC3 is upregulated in the UUO-induced renal fibrosis mouse model. The knockout of the HDAC3 gene does not affect the inflammatory regulator NF-κB p65 cytoplasm-to-nucleus translocation and accumulation in nuclei, but enhances the acetylation of NF-κB p65 at K122, decreases the transcriptional activity of NF-κB p65, and reduces the expression of pro-inflammatory factors (TNF-α, IL-1β, and IL-6). Additionally, the deficiency and inhibition of HDAC3 reduces the profibrotic gene expression and decreases the number and activation of myofibroblasts, ultimately alleviating UUO-induced renal fibrosis (Wang et al., 2024b). The increased expression of HDAC3 is observed in an UUO/aristolochic acid nephropathy (AAN)-induced renal fibrosis mice model. The inhibition of HDAC3 mitigates the abnormal expression of α-smooth muscle actin (α-SMA), collagen-1, E-cadherin, and bone morphogenetic protein 7 (BMP-7) to mitigate tubular injury and interstitial fibrosis induced by UUO/AAN treatment. Furthermore, HDAC3 forms inhibitory complexes with nuclear receptor corepressor (NCoR) and NF-κB, inhibiting the expression of Klotho, ultimately exacerbating renal fibrosis. Inhibiting HDAC3 can enhance the transcription of Klotho and reverse the abnormal downregulation of Klotho in fibrotic kidneys to alleviate renal fibrosis induced by UUO/AAN (Chen F. et al., 2021).

##### 3.2.1.4 HDAC4

**Diabetic kidney disease.** The expression of HDAC4 is upregulated in podocytes from DKD patients and podocytes cultured by HG *in vitro*. The overexpression of HDAC4 upregulates the expression level of calcineurin to increase the Bax expression and decrease the Bcl-2 expression, leading to the increased apoptotic rate of podocytes. The use of the pharmacological inhibitor of calcineurin can reverse the podocyte apoptosis caused by HDAC4 overexpression, a process associated with a decrease in Bax expression and increase in Bcl-2 expression (Shi et al., 2020). The HDAC4 and the long non-coding RNA small nucleolar RNA host gene 14 (SNHG14) levels are elevated in serum of DKD patients and HG-treated HK-2 cells. The overexpression of SNHG14 inhibits the expression of miR-483-5p to promote the HDAC4 expression, ROS production, and the inflammatory factor and ECM protein secretion, ultimately exacerbating HG-induced DKD (Huang et al., 2024).

**Renal fibrosis.** The expression of HDAC4 is upregulated in the UUO-induced renal fibrosis mice model. The inhibition of HDAC4 mitigates the partial epithelial–mesenchymal transition (pEMT) and G2/M phase cell cycle arrest after UUO, suppressing the expressions of fibronectin, α-SMA, and TGF-β to alleviate renal fibrosis. The UUO treatment induces the phosphorylation of Smad3, signal transducer and activator of transcription 3 (STAT3), and extracellular signal-regulated kinase 1/2 (ERK1/2) and upregulates their protein expression. However, the knockout and inhibition of HDAC4 reduces their phosphorylation and exerts anti-fibrotic effects through regulating the Smad3/STAT3/ERK1/2 pathway. In addition, the specific inhibition of HDAC4 also alleviates renal tubular injury and apoptosis induced by UUO. Mechanically, inhibiting HDAC4 reduces the expressions of NGAL (a biomarker of renal injury), Bax, and cleaved caspase-3 expression, restoring the Klotho expression, thereby exerting renoprotective and anti-fibrotic effects (Shen et al., 2022a).

##### 3.2.1.5 HDAC5

**Diabetic kidney disease.** HDAC5 is significantly upregulated in an UUO-induced renal fibrosis mice model and HK-2 cells treated with HG. The knockdown of HDAC5 increases the expression of E-cadherin and decreases the α-SMA expression to improve HG-induced endothelial-to-mesenchymal transition (EMT), thereby mitigating renal fibrosis by downregulating TGF-β1 expression. Additionally, the HDAC5 expression is regulated by the PI3K/Akt pathway. The blocking of the PI3K/Akt signaling pathway reduces the expression of HDAC5 and decreases the activity of TGF-β1, thereby inhibiting the EMT process in HG-treated HK-2 cells (Xu Z. et al., 2021). Additionally, HDAC5 induces Smad7 silencing by interacting with myocyte enhancer factor 2A (MEF2A) to inhibit the binding of MEF2A to the Smad7 promoter, leading to hypertrophic scar formation and fibroblast activation. However, the depletion of HDAC5 inhibits hypertrophic scar formation and fibroblast activation. Additionally, the knockdown of HDAC5 reduces TGF-β1-induced phosphorylation of Smad2/3 and increases Smad7 expression by interacting with MEF2A (Gao et al., 2022). This provides a novel therapeutic strategy for the treatment of renal fibrosis.

**Renal fibrosis.** The expressions of HDAC5 and G protein-coupled receptor kinase 5 (GRK5) are upregulated in UUO-induced renal fibrosis mice model and HK-2 cells treated with TGF-β. The GRK5, a serine/threonine kinase that can regulate G protein-coupled receptor (GPCR) signaling, is the upstream regulator of the HDAC5/Smad3 signal pathway. The inhibition of GRK5 can regulate the HDAC5/Smad3 pathway to alleviate renal fibrosis. Specifically, GRK5 upregulates HDAC5 expression and interacts with myocyte enhancer factor 2A (MEF2A) to suppress MEF2A’s transcriptional activity, thereby leading to reduced Smad7 transcription and enhanced Smad3 activation, ultimately promoting fibrotic progression. Notably, the inhibition of GRK5 effectively downregulates HDAC5 expression, alleviating renal fibrosis and injury in a model of UUO and TGF-β-induced fibrosis (Xiang et al., 2024).

##### 3.2.1.6 HDAC6

**Diabetic kidney disease.** HDAC6 is activated in the type 2 diabetic mice, the db/db mice, and the podocytes exposed to AGE. The HDAC6 inhibition protects db/db mice kidneys from hyperglycemia. HDAC6 partially governs autophagy by deacetylating α-tubulin at K40, leading to microtubule depolymerization, reducing autophagy, and increasing podocyte motility under AGE stimulation, ultimately contributing to DKD progression. The regulatory role of HDAC6 in podocytes autophagy and motility indicates that HDAC6 functions as a DKD treatment target (Liang et al., 2020). Furthermore, the HDAC6 expression is upregulated in the RTECs of DKD patients, the STZ-induced DKD mice model, and HK-2 cells treated with TGF-β. The inhibition of HDAC6 reduces macrophage infiltration, tubular damage, and tubular interstitial fibrosis by suppressing the expression of collagen I and α-SMA induced by TGF-β. Additionally, the inhibition of HDAC6 suppresses the activation of NLRP3, caspase-1, and ASC induced by TGF-β to alleviate pyroptosis (Hou et al., 2022).

**Polycystic kidney disease.** The occurrence of ADPKD is primarily attributed to mutations in the Pkd1 gene or Pkd2 gene, which, respectively, encode polycystin 1 (PC1) and polycystin 2 (PC2) (Luo et al., 2023a). The expression and activity of HDAC6 are elevated in Pkd1-mutant mouse embryonic kidney (MEK) epithelial cells. By inhibiting the expression of HDAC6, the acetylation level of α-tubulin is enhanced and the expression of epidermal growth factor receptor (EGFR) in Pkd1-mutant renal epithelial cells is reduced, which significantly decreases the formation of renal cysts and improves renal function. Moreover, the inhibition of HDAC6 reduces the phosphorylation of ERK1/2 (the downstream target of EGFR), normalizing the localization of EGFR from the apical to the basolateral side in the kidneys of Pkd1 knockout mice, delaying cyst formation. Based on this mechanism, targeting HDAC6 to reduce EGFR activity may become a potential therapeutic strategy for the treatment of PKD (Liu et al., 2012). In the Pkd1-mutated mice model, the upregulation of PC2 expression is a common phenomenon, which is partly due to the increase in Pkd2 mRNA levels. The HDAC6-glucose-regulated protein 94 (GRP94) axis plays a significant role in maintaining the elevated levels of PC2 in Pkd1-mutant cells, thereby affecting the progression of the disease. By inhibiting HDAC6 activity, the activation of HSP90 protein can be reversed, which in turn suppresses the function of GRP94 (an ER protein and a member of the HSP90 chaperone family), reducing PC2 levels, ultimately preventing the formation of cysts (Yao et al., 2021).

##### 3.2.1.7 HDAC9

**Diabetic kidney disease.** The expression of HDAC9 is upregulated, and the expression of miR-383 is downregulated in DKD patients with type 2 diabetes, accompanied by mesangial dilation, podocyte loss, and albuminuria. Notably, miR-383 can inhibit HDAC9 to reduce podocyte injury and glomerular sclerosis. Mechanistically, miR-383 inhibits HDAC9 expression and activity by directly targeting the complementary binding sites located in the 3′ untranslated region (3′ UTR) of HDAC9. This suppression reduces ROS production and podocin/nephrin expression, ultimately mitigating podocyte damage through mediating the JAK2/STAT3 pathway. Based on this mechanism, the inhibition of HDAC9 also reduces the release of inflammatory factors and alleviates inflammation (Fan W. et al., 2020).

*Renal fibrosis.* The increased expression of HDAC9 is observed in an UUO/AAN-induced renal fibrosis male mouse model. The specific knockout or inhibition of HDAC9 can alleviate G2/M phase arrest in renal epithelial cells, thereby reducing the expressions of pro-fibrotic cytokines (collagen I, collagen IV, vimentin, α-SMA, and TGF-β1), ultimately alleviating renal tubular injury and tubulointerstitial fibrosis. Mechanically, HDAC9 promotes the occurrence of tubulointerstitial fibrosis by deacetylating STAT1 and promoting its reactivation, resulting in G2/M phase arrest in RTECs. By inhibiting or knockout HDAC9, this pathological process can be significantly reversed, and renal fibrosis induced by UUO/AAN can be effectively improved (Zhang et al., 2023c).

#### 3.2.2 SIRTs in chronic kidney disease

##### 3.2.2.1 SIRT1

**Diabetic kidney disease.** Yin yang 1 (YY1), a zinc finger transcription factor, plays a regulatory role in renal fibrosis. The decrease of SIRT1 in db/db mice and HG-treated HK-2 cells enhances YY1 acetylation and EMT and accelerates fibrosis. Additionally, the knockdown of YY1 decreases the protective effects of resveratrol (an agonist of SIRT1) on EMT and fibrosis in db/db mice (Du et al., 2021). The expression of SIRT1 is reduced in the STZ-induced diabetes mice model and the HK-2 cells treated with HG, accompanied by the dilation and atrophy of renal tubules, the absence of brush-like boundaries, and the formation of tubular shapes. SIRT1 is involved in the protective mechanism of polysulfide donor Na_2_S_4_ against HG-treated HK-2 cell damage. Mechanistically, Na_2_S_4_ increases the expression of SIRT1, reducing the phosphorylation and acetylation levels of NF-κB p65 and STAT3 and inhibiting the Bax, cleaved caspase-3, and cleaved PARP protein expressions to reduce apoptosis. Additionally, Na_2_S_4_ inhibits the inflammatory cytokine expression and ROS production to alleviate renal dysfunction and histological damage (Sun et al., 2021). The SIRT1 expression is decreased in the high-fat diet (HFD)-induced DKD mice model and HG-treated SV40 MES13 cells, accompanied by the increased thioredoxin-interacting protein (Txnip) expression and nuclear translocation of XBP1s (a transcription factor that binds to Txnip promoters). Exendin-4 (the glucagon-like peptide 1 receptor agonist) significantly increases SIRT1 expression, decreasing Txnip expression and XBP1s nuclear translocation through suppressing the acetylation level of H3K9 in the Txnip promoter region mediated by SIRT1, ultimately improving the renal pathological injury induced by HFD/HG. The absence of SIRT1 prevents exendin-4 from downregulating Txnip expression, further reducing the protective effects of exendin-4 on HFD-/HG-induced renal pathological injury (Wang M. J. et al., 2023). The expression of SIRT1 is downregulated, but the expressions of LncRNA AK044604 (insulin sensitivity and autophagy regulatory factor) and the activated glycogen Synthase Kinase 3 beta (GSK3β) are increased in db/db mice and HG-treated immortalized mouse podocyte cells (MPCs). In the STZ-induced DKD rat model and AGES-treated human umbilical vein endothelial cells (HUVECs), the expression of SIRT1 is significantly downregulated, while the mRNA levels of LC3 and beclin 1 are significantly decreased. Resveratrol, as a SIRT1 agonist, can significantly upregulate the expressions of SIRT1, beclin 1 and LC3II/I, while down-regulating the expression of p62, thereby promoting the autophagic activity of HUVECs. In addition, resveratrol can also significantly reduce the levels of endothelial injury factors, including AGEs, Col Ⅳ, ET-1, e-selectin, fibronectin high-sensitivity c-reactive protein (hs-CRP), intercellular adhesion molecule-1 (ICAM-1), matrix metalloproteinase-2 (MMP2), nitric oxide (NO), and vascular cell adhesion molecule-1 (VCAM-1). Notably, the effect of resveratrol on the expression of beclin 1 and SIRT1 is significantly weakened, while the level of autophagy in cells decreased after the knockout of the SIRT1 gene (Liu et al., 2024b). The upregulation of LncRNA AK044604 expression reduces the autophagy levels by inhibiting SIRT1 expression, thereby exacerbating podocyte damage. Conversely, the inhibition of LncRNA AK044604 upregulates SIRT1 expression and enhances SIRT1-induced autophagy, further alleviating podocyte damage (Su et al., 2022). Additionally, lncRNA HOXB3OS ameliorates HG-induced podocyte damage. HOXB3OS binds to Ythdc2 and inhibits the binding of Ythdc2 to SIRT1 mRNA, thereby suppressing the degradation of SIRT1 mRNA, reducing podocyte loss and delaying the progression of DKD (Wang et al., 2025e). Many edible and medicinal plants can improve DKD through protecting renal mitochondrial function, regulating ERS, anti-inflammation, reducing oxidative stress, and protecting podocytes. For example, puerarin, the main active component of kudzu root, can activate the SIRT1/NF-κB pathway to inhibit podocyte oxidative stress, enhance renal autophagy, and alleviate glomerular damage. Furthermore, puerarin may restore autophagy and inhibit podocyte apoptosis through the heme oxygenase-1 (HMOX-1), SIRT1, and AMPK pathways, thereby improving renal function. Geniposide, the main active ingredient of *Gardenia*, can alleviate oxidative stress and inflammatory responses in DKD through the AMPK/SIRT1/NF-κB pathway. Bavachin is a flavonoid extracted from *Psoralea corylifolia L*, which can improve the renal function of db/db mice and alleviate renal fibrosis. At the molecular level, bavachin increases the protein expression of PGC-1α, SIRT1, nuclear respiratory factor 1 (Nrf1), and mitochondrial transcription factor A (mtTFA) in DKD mice, restores the damaged mitochondrial morphology, enhances mitochondrial biosynthesis, and reduces the number of abnormal mitochondria (Yan et al., 2025).

SIRT1 expression is downregulated in the pod-HIV mouse model. The pod-HIV mouse model is characterized by the relatively low expression of HIV-1 proviral genes in podocytes, and exacerbation of podocyte damage has been observed in the model. The decrease in SIRT1 expression is a key factor in the progression of DKD in patients with HIV and diabetes. Mechanistically, diabetes and HIV-1 collaboratively increase the expression of miR-34a in the glomeruli, leading to the reduction in SIRT1 expression. These changes are also associated with the increased acetylation level of p53 and NF-κB p65 and the enhancement in the expression of aging and inflammatory markers. The treatment with the specific SIRT1 activator BF175 in diabetic pod-HIV mice can effectively alleviate albuminuria and glomerular lesions (Feng et al., 2021).

Additionally, the activation of the SIRT1/peroxisome proliferator-activated receptor γ coactivator-1α (PGC-1α) pathway attenuates RTECs injury in the RIRI mice model (Feng et al., 2023).

**Hypertensive nephropathy.** Renovascular hypertension is one of the most common causes of secondary hypertension and often leads to refractory hypertension. It is defined as systemic hypertension secondary to impairment of the renal blood supply (Nair and Vaqar, 2025). If left unaddressed over the long term, renal vascular hypertension may potentially progress to HTN. Relevant studies indicate that the progression of renal vascular hypertension is closely associated with the decreased expression of SIRT1 protein. In the heart and kidneys, the reduction in SIRT1 expression and the systemic or local effects of angiotensin II may be significant factors in the development of hypertension (Yeganeh-Hajahmadi et al., 2021). A combination of SIRT1 activators and angiotensin II receptor blockers for the treatment of HTN may be a good strategy. The expression of SIRT1 and FoxO3a is significantly reduced in a spontaneous hypertensive rats (SHR) model and the NRK-52E cells exposed to Ang II. Cordyceps can reduce hypertensive renal fibrosis in the SHR model and Ang II-treated NRK-52E cells, which is dependent on the regulation of SIRT1. Specifically, cordyceps inhibits oxidative stress and reduces ECM accumulation and autophagy, thereby inhibiting renal fibrosis and improving hypertensive renal injury by upregulating SIRT1/Fox3a expression (Cai et al., 2021). The SIRT1 expression is downregulated in the SHR model, accompanied by increased ROS. Jiangya Tongluo decoction alleviates pathological changes of renal tissue in SHRs, including glomerular ischemia and sclerosis, interstitial fibrosis, inflammatory cell infiltration, and compensatory dilation of renal tubules. Mechanistically, JYTL inhibits SHRs mitochondrial dysfunction and promotes mitochondrial biogenesis by upregulating the expressions of SIRT1, PGC-1α, Nrf1, and transcription factor A, mitochondrial (TFAM), thereby alleviating the pathological changes of SHRs. In addition, mitochondrial autophagy mediated by threonine-protein kinase (PINK1)/E3 ubiquitin-protein ligase parkin (parkin) is promoted by upregulation of SIRT1 expression. Importantly, SIRT1 inhibitors reduce the protective effect of JYTL on renal mitochondrial dysfunction by inhibiting the expression of SIRT1 (Zhao et al., 2024). The decreased expression of SIRT1/PGC-1α has been observed in the deoxycorticosterone acetate (DOCA)-salt induced hypertension rat model treated with the 5/6 nephrectomized mouse model and H_2_O_2_-treated NRK-52E cells. The H_2_O_2_ treatment inhibits the activity of NRK-52E cells and aggravates the oxidative stress response. Notably, the oxidative stress compromises tubular cell and parenchymal integrity and deteriorates renal function by downregulating SIRT1/PGC-1α-Mfn2 signaling. The use of dapagliflozin and entresto effectively protects renal function and maintains renal structural integrity by regulating SIRT1/PGC-1α-Mfn2 and cellular stress signaling (Ko et al., 2023).

**Polycystic kidney disease.** The expressions of SIRT1 and cellular MYC proto-oncogene protein (c-MYC) are upregulated in Pkd1 mutant renal epithelial cells and tissues. The knockout and inhibition of c-MYC reduces the expression of SIRT1 through binding to two potential c-MYC–binding sites (E-boxes E1 and E2) of the SIRT1 promoter. In addition, TNF-α detected in the cyst fluid promotes cyst formation and induces the expression of SIRT1 mRNA and protein by activating the NF-κB pathway in Pkd1-deficient MEK cells and PN24 cells. However, NF-κB inhibitors effectively block the upregulation of SIRT1 expression induced by TNF-α. The inhibition of SIRT1 can increase the acetylation level of retinoblastoma protein (Rb) in Pkd1-deficient MEK cells while decreasing its phosphorylation level, releasing E2F transcription factor 1 (E2F1) from the RB-E2F complex, regulating the proliferation signal of renal cystic epithelial cells, and delaying the growth of cysts in ADPKD patients, thereby increasing the apoptosis of cystic epithelial cells (Zhou et al., 2013). Based on the important role of SIRT1 in ADPKD, niacinamide as a SIRT1 inhibitor has also been shown to delay cyst formation in ADPKD patients through inhibiting the SIRT1 expression, thereby protecting renal function (El Ters et al., 2020).

##### 3.2.2.2 SIRT2

**Diabetic kidney disease.** The expression of SIRT2 is upregulated in the murine renal podocytes MPC5 treated with HG and SIRT2 reduces cell proliferation and apoptosis by reducing the level of autophagy in glomerular podocytes. However, the knockdown of SIRT2 results in significant enhancement of cell proliferation activity and decreased apoptosis. Therefore, SIRT2 may be a causative factor of DKD and lead to disease progression (Liu S. et al., 2022). The SIRT2 expression is upregulated in the renal tissue of HFD-/STZ-induced DKD mice and in the HG-/palmitic acid (PA)-treated HK-2 cells. The knockout of the SIRT2 gene effectively alleviates kidney injury induced by HFD/STZ treatment, including glomerular and mesangial stroma enlargement, renal tubule dilation, and inflammatory responses induced by HG/PA, whereas the overexpression of SIRT2 exacerbates these responses. The underlying mechanism is related to the increase in the level of c-Jun/c-Fos (two transcription factors, which play a crucial role in inflammation) acetylation in RTECs, accompanied by the interaction between c-Jun phosphorylation and acetylation. The inhibition of SIRT2 increases the acetylation level of c-Jun/c-Fos and decreases the binding activity of AP-1 (a complex formed by activated C-Jun and c-Fos) and the inflammatory factor MCP-1, thereby alleviating the HG/PA-induced inflammatory response (Chen et al., 2025).

Renal fibrosis is the main pathological feature of DKD. Interestingly, the SIRT2 expression is reduced in the renal tubulointerstitial tissue of DKD patients and UUO-induced renal fibrosis mice. The specific absence of SIRT2 in RTECs aggravates renal fibrosis in UUO mice, while SIRT2 overexpression has the opposite effect. SIRT2 deacetylates Smad2 and promotes its ubiquitination at K451, deacetylates Smad3 at K341 and K378, and reduces the phosphorylation, acetylation, and nuclear translocation of Smad2 and Smad3 in the kidneys of UUO mice to inhibit the activation of Smad2 and Smad3, ultimately mitigating kidney injury in the UUO mice model (Yang et al., 2023).

**Hypertensive nephropathy.** The expression of SIRT2 is upregulated in an Ang II-induced HTN mice model. Septin4 is a pro-apoptotic protein, and the 174th amino acid (K174) site of septin4 is a key regulatory point for hypertensive kidney injury. SIRT2 interacts with the GTPase domain of septin4 and deacetylates K174 on septin4, suppressing the cleaved caspase-3 and cleaved-PARP1 pathway, further alleviating Ang II-induced hypertensive kidney injury and glomerulosclerosis. However, the knockout of SIRT2 contributes to the increased acetylation level of septin4-K174 and aggravates oxidative stress and kidney injury in Ang II-induced HTN mice (Dikalov and Kirabo, 2023; Zhang et al., 2023d).

##### 3.2.2.3 SIRT3

**Diabetic kidney disease.** The expression of SIRT3 and nicotinamide phosphoribosyl transferase (Nampt) is decreased in the kidney of BTBR ob/ob mice. The activation of SIRT3 can reduce albuminuria, glomerular injury, and podocyte injury. The protective effect of SIRT3 in glomerulopathy is related to the increased levels of SIRT3 and Nampt. Specifically, the upregulation of SIRT3 and Nampt in renal tubular cells provides the NMN (an intermediate of NAD^+^ synthesis) to diabetic podocytes and other glomerular cells, increasing glomerular NAD^+^ and SIRT3 activity, thereby alleviating proteinuria and glomerular injury in BTBR ob/ob mice (Locatelli et al., 2020). The expressions of SIRT3, beclin-1, and LC-3II are downregulated in HG-treated HK-2 cells, accompanied by the activation of the Notch homolog 1 (Notch-1)/hairy and enhancer of split-1 (Hes-1) pathway. The increased expression of SIRT3 counteracts the suppression of beclin-1 and LC-3II expression, the increase in p62 levels, and the upregulation of the Notch-1/Hes-1 signaling pathway induced by HG to promote autophagy, thereby mitigating DKD (Wang et al., 2021).

SIRT3 is indispensable in the pathological process of DKD affected by the (Pro)renin receptor (PRR). In the renal tubules, the expression level of PRR is high, and knocking out PRR can effectively reduce pyroptosis and interstitial inflammation in the RTECs of db/db mice, significantly decreasing kidney damage. At the mechanistic level, PRR specifically binds to the cysteine-rich region at the C-terminus of DPP4, increasing the level of dipeptidyl peptidase 4 (DPP4) protein, which in turn activates the JNK signaling pathway while inhibiting SIRT3 and fibroblast growth factor receptor 1 (FGFR1) signals, leading to the death of pyroptotic cells (Xie S. et al., 2024).

The expressions of SIRT3 and meteorin-like (Metrnl) are decreased in STZ/HFD-induced DKD mice and PA-treated RTECs. The overexpression of metrnl induces the expression of SIRT3 by promoting the expression of transcriptional activator PGC-1α, increasing the expression level of UCP1 and the activation of AMPK in PA-treated RTECs to accelerate mitochondrial autophagy, maintain mitochondrial homeostasis, and alleviate lipid accumulation (a thermogenic factor) (Zhou et al., 2023). SIRT3 is important in the protective mechanism of CD38 deficiency against HFD/STZ-induced DKD mice. The CD38 deficiency prevents DKD by activating the SIRT3 pathway to inhibit renal fibrosis, lipid accumulation, and oxidative stress (Wang L. F. et al., 2025). The expression of apelin (an endogenous ligand of apelin/apelin receptor (APJ) can alleviate endothelial cell dysfunction in DKD) is increased in STZ-induced diabetic mice, but the expression of APJ is decreased. In addition, apelin/APJ increases the expression of SIRT3 and KLF15 (an anti-fibrotic transcription factor), promotes the deacetylation and translocation of KLF15 to the nucleus, and prevents the synthesis of laminin and collagen IV in glomerular endothelial cells (GECs), ultimately reducing the thickening of GBM in early DKD (Huang M. et al., 2025).

**Hypertensive nephropathy.** The expression of SIRT3 is downregulated in high salt diet (HSD)-induced dahl salt-sensitive rats hypertensive renal injury model and Ang II-treated HK-2 cells. HSD induces the oxidative stress and promotes glomerular and tubulointerstitial fibrosis. Notably, canagliflozin can restore SIRT3 expression, reducing oxidative stress and renal fibrosis, therefore improving kidney damage. Specifically, canagliflozin exerts its protective effect on the kidneys by regulating the SIRT3/FoxO3a/catalase pathway to reduce oxidative stress, inhibiting EMT in the HSD-induced dahl salt-sensitive rat hypertensive renal injury model and Ang II-treated HK-2 cells. However, the deficiency of SIRT2 counteracts the protective effects of canagliflozin on hypertensive renal injury *in vitro* and vivo (Wang Z. et al., 2023; Zhang et al., 2023d).

##### 3.2.2.4 SIRT4

**Diabetic kidney disease.** The expression of SIRT4 is downregulated in DKD patients and HG-treated podocytes. Forkhead box M1 (FOXM1) binds to the SIRT4 promoter to induce SIRT4 transcriptional activation, reduce the expression of pyroptosis-associated NLRP3 inflammasome and cleaved caspase 1, and increase the activity of HG-treated podocytes. However, the inhibition of SIRT4 counteracts the protective effects of FOXM1 on the kidneys of STZ-induced DKD mice, including the glomerular mesangial matrix hyperplasia and glycogen accumulation in glomeruli in tissue (Xu X. et al., 2021). The downregulation of SIRT4 and miR-124-3p expression is observed in the HG-/HFD-induced db/db mice, while the forkhead box Q1 (FOXQ1) expression is upregulated. In addition, granular tubular epithelial cells with vacuolar degeneration and impaired renal function are observed in db/db mice. The use of miR-124-3p inhibitors inhibits miR-124-3p levels, leading to the increased expression of FOXQ1. The FOXQ1 binds gene promoters to SIRT4 and inhibits SIRT4 transcription. The reduction of SIRT4 affects mitochondrial fusion and fission and reduces ATP synthesis, leading to mitochondrial dysfunction and the large amounts of ROS, thereby enhancing renal oxidative stress in db/db mice and promoting the development of DKD (Liang et al., 2023).

**Renal fibrosis.** The elevated nuclear accumulation of SIRT4 is observed in the RTECs of the UUO/IRI/FA-induced renal fibrosis mice model. The deficiency of SIRT4 in RTECs significantly mitigates fibrotic effects in these renal fibrosis models. Specifically, SIRT4 interacts with U2 small nuclear RNA auxiliary factor 2 (U2AF2) at the K413 site under TGF-β 1 stimulation or renal injury to deacetylate U2AF2, promoting cellular communication network 2 (CCN2, a marker of fibrosis activity) expression through alternative pre-mRNA splicing, ultimately contributing to the progression of kidney fibrosis. Notably, the gene knockout or inhibition of SIRT4 reverses these changes, therefore mitigating renal fibrosis induced by UUO/IRI/FA treatment (Yang et al., 2024).

##### 3.2.2.5 SIRT6

**Diabetic kidney disease.** The pathogenesis and progression of DKD are closely associated with disorders of glucose and lipid metabolism. SIRT6 optimizes the metabolic state of glucose and lipids by controlling glycolysis and gluconeogenesis, affecting insulin secretion and action, and regulating lipid catabolism, transport, and synthesis. Therefore, SIRT6 plays a key role in regulating glucose and lipid metabolism and signaling, particularly in DKD (Wang et al., 2023c). The SIRT6 expression is downregulated in HG-induced db/db mice and the glomerular podocytes treated by HG, accompanied by the increased glomerular volume, compensatory renal tubule hypertrophy, inflammatory cell infiltration, and the increased mesangial matrix. SIRT6 alleviates mitochondrial dysfunction, podocyte injury, and ferroptosis induced by HG through activating the Nrf2/GPX4 pathway and increasing SLC7A11, HO-1, and SOD2 expressions. Notably, the inhibition of the Nrf2/GPX4 signaling pathway reduces the protective effects of SIRT6 on HG-treated glomerular podocytes (Du et al., 2024). The expressions of SIRT6, PI3K, and Akt are downregulated in db/db mice and HG-treated renal podocytes. Cytoskeletal remodeling is an early manifestation of podocyte injury in DKD. SIRT6 can protect podocytes from HG-induced cytoskeletal remodeling and apoptosis by activating the PI3K/Akt signaling pathway. However, the treatment of the SIRT6 inhibitor aggravates HG-induced podocyte injury by exacerbating cytoskeletal remodeling and apoptosis in HG-treated podocytes. The molecular mechanism of how SIRT6 activates the PI3K/Akt pathway and the interaction between SIRT6 and PI3K/Akt pathway are still unclear (Zhang Z. et al., 2024). Additionally, the specific deletion of SIRT6 leads to the activation of the Notch1/Jagged1 (Jagged1 is a ligand in the Notch signaling pathway, which activates the Notch signaling pathway by binding to the Notch receptor) signaling pathway, exacerbating the inflammatory response and autophagic damage of podocytes in the HG-treated podocytes. The Notch signaling pathway can promote the activation of T cells, leading to cell proliferation, autophagy, cell recruitment, proteolysis, and inflammatory response. The increased expression of SIRT6 can effectively inhibit the Notch1/Jagged1 signaling pathway, reducing the secretion of pro-inflammatory factors including IL-6 and TNF-α, thereby significantly alleviating DKD (Ma et al., 2025).

The expression of SIRT6 is reduced in STZ-induced DKD mice and sunitinib-induced nephropathy model. Notch controls signaling communication between adjacent cells and plays a fundamental role in kidney development. Nod-like receptor family, pyrin domain-containing 3 (NLRP-3) is kind of inflammatory mediator. The specific deletion of SIRT6 activates the transcription of the transmembrane Notch receptors Notch1 and Notch4, reducing the anti-inflammatory and anti-apoptotic effects of SIRT6 in podocytes and exacerbating podocyte damage and proteinuria. However, the upregulation of SIRT6 inhibits the transcription of Notch1 and Notch4 through deacetylating H3K9 and significantly downregulates the secretion of NLRP3 and the pro-inflammatory factor IL-1β, thereby alleviating the inflammatory response, mitigating podocyte damage and glomerulosclerosis (Liu et al., 2017; Elrashidy et al., 2024).

**Hypertensive nephropathy.** SIRT6 effectively prevents the occurrence of hypertension and its complications by regulating endothelial cell function. The SIRT6 expression is downregulated in the kidney of hypertensive mouse model treated with deoxycorticosterone acetate/salt and induced by Ang II. The specific loss of SIRT6 in endothelial cells significantly increases blood pressure and exacerbates endothelial dysfunction and kidney damage under hypertensive conditions. Mechanically, SIRT6 binds to the promoter of Nkx3.2 (NK3 homeobox2) and inhibits the transcription of Nkx3.2 by suppressing H3K9ac and H3K56ac in the promoter of Nkx3.2 to prevent endothelial damage. Additionally, SIRT6 induces the expression of GATA-binding protein 5 (GATA5, a novel blood pressure regulator) and regulates the GATA5-mediated signaling pathway to prevent hypertension and its complications caused by endothelial dysfunction and improve apoptosis and mitochondrial dysfunction induced by Ang II (Guo et al., 2019). The SIRT6 expression is reduced in Ang II-treated mouse podocytes. The specific knockout of SIRT6 induces the formation of lipid droplets and exacerbates Ang II-induced glomerular podocyte loss and cholesterol accumulation. In addition, the loss of SIRT6 reduces the protective effects of CD (cholesterol-lowering drugs) against Ang II-induced podocyte damage *in vitro* and *vivo*. Ang II treatment reduces the ABCG1 (an important molecule associated with cholesterol homeostasis) expression, and the overexpression of SIRT6 improves Ang II-induced lipid droplet formation, cholesterol accumulation, and cholesterol efflux capacity, restoring ABCG1 expression, further affecting ABCG1-mediated cholesterol efflux to ameliorate Ang II-induced podocyte injury (Yang et al., 2020). The overexpression of SIRT6 inhibits ROS production and DNA double-strand breaks (DNA DSB) in the podocytes exposed to Ang II, alleviating Ang II-induced podocytes damage. In addition, the overexpression of SIRT6 upregulates the decreased expression of Nrf2 and HO-1 induced by Ang II. The knockdown of Nrf2 reduces the protective effect of SIRT6 against Ang II-induced podocyte damage (Fan et al., 2021).

##### 3.2.2.6 SIRT7

**Diabetic kidney disease.** The significant upregulation of SIRT7 and miR-20b in HG-treated podocytes has been shown to inhibit podocyte apoptosis, and the miR-20b inhibitors can promote the expression of SIRT7, thereby reducing HG-induced apoptosis. Specifically, SIRT7 is a target of miR-20b, and the inhibition of miR-20b increases the mRNA and protein expression of SIRT7 by direct targeting of the 3′-UTR of SIRT7 in HG-treated podocytes, alleviating HG-induced podocyte apoptosis (Qi et al., 2022a). The SIRT7 expression is decreased in the kidneys of HG-treated GECs and STZ-induced DKD mice, accompanied by the increased α-SMA expression and decreased CD31 expression. In addition, HG treatment increases the fascin level in GECs and induces the process of endothelial-to-mesenchymal transition (EndMT). The increased expression of SIRT7 reduces the expression of fascin by binding to the promoter of fascin, thereby inhibiting EndMT, reducing kidney damage and fibrosis, ultimately improving renal function in DKD mice (Wu M. et al., 2024). Additionally, even when blood sugar levels are maintained within the normal range, the phenomenon of EndMT can still occur, and the role of the SIRT7/hypermethylated in the cancer 1 (HIC1)/syndecan-1 (SDC1) signaling pathway in the EndMT process should not be underestimated. The expressions of SIRT7 and SDC1 are decreased in DKD patients, diabetic renal injury rat model, and HG-treated HGECs, accompanied by collagen deposition and interstitial fibrosis. Notably, the decreased expression of SIRT7 remains after the normalization of blood glucose. SIRT7 promotes the expressions of SDC1 and CD31 in GECs by binding with HIC1, decreases the expression of α-SMA, and alleviates EndMT to play a role in metabolic memory and DKD (Lu L. et al., 2024).

**Hypertensive nephropathy.** The SIRT7 expression is downregulated in an Ang II-induced hypertensive mouse model. Decreased expressions of HO-1 and NQO1 (the downstream targets of Nrf2) and significantly decreased GPX4 expression are observed in hypertensive mice. The overexpression of SIRT7 significantly reduces ferroptosis and lipid peroxidation in the kidneys of hypertensive mice. Specifically, SIRT7 enhances the Nrf2/GPX4/xCT (cystine/glutamate antiporter) and HO-1/NQO1 signaling pathways, thereby restoring the antioxidant capacity of the kidneys. In addition, the upregulation of SIRT7 expression significantly improves renal EMT and subsequent renal fibrosis in hypertensive mice and reverses the decreased KLF15 expression induced by Ang II. In conclusion, SIRT7 plays a key role in alleviating kidney dysfunction and damage during high blood pressure through promoting KLF15/Nrf2, HO-1/NQO1 and xCT/GPX4 signaling pathways (Li X. T. et al., 2022).

## 4 The renoprotection of SIRTs and HDACs activators and inhibitors

### 4.1 Activators

SIRTs are an evolutionarily highly conserved family of deacetylases and dependent on NAD^+^. Seven members (SIRT1–SIRT7) have been found in mammals. These enzymes are widely involved in the regulation of many important biological processes including energy metabolism, oxidative stress, inflammation, apoptosis and aging by regulating the acetylation modification state of substrate proteins (Wu et al., 2022). The expression level and activity changes of SIRTs can regulate the acetylation of key proteins and play an important role in kidney development and physiological function. These effects involve multiple biological processes, including mitochondrial energy metabolism, oxidative stress balance, autophagy regulation, DNA damage repair, inflammatory response, fibrotic processes, and apoptosis. It is worth noting that the changes in SIRTs activity and expression are closely related to the pathophysiological processes of kidney disease, including AKI and CKD (Clark and Parikh, 2021; Perico et al., 2024). Because of the critical role of SIRTs in kidney protection, their agonists are potential therapeutic agents. At present, the most studied SIRT agonists mainly target SIRT1, SIRT3, and SIRT6, and these activators upregulate the expression and activity of these enzymes to mediate the related pathways, thus playing a protective role in the kidney (Table 3). Therefore, we mainly discuss these agonists that target SIRT1, SIRT3, and SIRT6.

**TABLE 3:** The mechanism of SIRTs activators in renal diseases.

Enzymes	Activators	Diseases		Pathway	Pathophysiological	References
SIRT1	Metformin	SAKI		MCPIP1/SIRT1/NF-κB	Inflammation and apoptosis	Zhang et al. (2024a)
	Resveratrol	DKD		JAML/SIRT1/SERBP-1/ChREBP	Lipid deposition	Gu et al. (2022)
	Quercetin	AKI	SAKI	SIRT1/NF-κB	Inflammation, apoptosis	Lu et al. (2021)
			CI-AKI	SIRT1/Nrf2/HO-1/SOD1	Oxidative stress, apoptosis, and inflammation	Wu et al. (2024b)
		HTN		SIRT1/PGC-1α/Nrf1/TFAM PINK1/Parkin	Fibrosis, inflammation, and autophagy	Zhao et al. (2024)
		Renal fibrosis		SIRT1/PINK1/Parkin	Autophagy and fibrosis	Liu et al. (2020b)
	Curcumin	AKI		SIRT1/FoxO1/p-65	Oxidative stress, apoptosis, and inflammation	Li et al. (2021c)
	Melatonin	AKI		SIRT1/NF-κB	Oxidative stress and DNA damage	Üstündağ et al. (2023)
		DKD		SIRT1/FoxO3a/AMPK/Nrf2	Autophagy and oxidative stress	Siddhi et al. (2022)
	SRT1720	Renal fibrosis		SIRT1/MDA/TGF-β1/CTGF/GSH/SOD/GPX	Inflammation and fibrosis	Ren et al. (2017)
SITR3	Honokiol	AKI		SIRT3/AMPK/Drp1	Apoptosis and mitochondrial fragmentation	Mao et al. (2022)
				SIRT3/LKB1/AMPKα	Fatty acid oxidation and deposition	Li et al. (2020)
		Renal fibrosis		SIRT3/PDHE1α/PDH	Fibrosis	Zhang et al. (2021b)
	Melatonin	AKI		SIRT2/TFAM/PINK1 PRKN/LC3-II	Mitophagy and inflammation	Deng et al. (2024)
				SIRT3/Nrf2/HO-1	Ferroptosis, inflammation, oxidative stress, and fibrosis	Qiu et al. (2022)
	Metformin	AKI		SENP1-SIRT3/AMPK/	Apoptosis, inflammation, and fibrosis	Zhu et al. (2023)
SIRT6	Daphnetin	AKI		SIRT6//Nrf2/HO-1/NQO1 NF-κB/MAPK/p53	Oxidative stress, inflammation, and apoptosis	Fan et al. (2020c)
	Isoorientin	AKI		SIRT6/Nrf2/HO-1/NF-κB/HMGB1/JNK/ERK/p38	Inflammation, apoptosis, and oxidative stress	Fan et al. (2020b)

#### 4.1.1 SIRT activators

##### 4.1.1.1 The activator of SIRT1

**Metformin.** Metformin, as a first-line antihyperglycemic medication, is widely used in the treatment of type 2 diabetes with obesity (Pan et al., 2020). Metformin can alleviate glomerular capillary dilation, renal interstitial edema, renal tubular cell injury, and apoptosis to mitigate LPS-induced SAKI by enhancing the MCPIP1/SIRT1 signaling pathway. Specifically, metformin increases the expressions of MCPIP1 and SIRT1 to inhibit the acetylation of NF-κB p65, further suppressing the NF-κB-mediated inflammatory responses, reducing inflammatory factor levels, and regulating the expression of related proteins in renal tissue (Zhang W. L. et al., 2024).

Metformin can reduce the thickness of GBM and foot process fusion in diabetic rats, improve oxidative stress and apoptosis and protect renal function by activating the SIRT1/FoxO1 autophagy signal axis to promote the autophagy level, and inhibit ECM secretion induced by HFD/STZ in diabetic rats. Mechanistically, metformin significantly upregulates the expressions of SIRT1 and FoxO1, the ratio of LC3-II/LC3-I, promotes the Beclin-1 expression, inhibits the expressions of p62, collagen I, collagen IV, and FN, and promotes autophagy to exert renal protective function (Xu et al., 2020).

Metformin can exert anti-inflammatory and kidney protective effects to effectively reduce crystal deposition and renal tubular injury in glyoxalate-induced calcium oxalate kidney stone disease (CaOx) nephrocalcinosis mouse model. Mechanistically, metformin enhances the expression and activity of SIRT1 and subsequently downregulates TLR4, p-NF-κB, IL-1β and iNOS (the makers of proinflammatory M2 macrophages) and upregulates Arg1 (the maker of anti-inflammatory M1 macrophages) expressions to increase M2 macrophage polarization and inhibit M2 macrophage polarization (Liu H. et al., 2023).

Although metformin has a certain protective effect in kidney diseases including AKI, studies have found that it has potential adverse reactions. Metformin may cause lactic acidosis, and its mechanism includes inhibiting the activity of mitochondrial complexes, leading to reduced ATP production, which in turn causes lactic acid accumulation and subsequently triggers lactic acidosis. This is also one of the main reasons for its limited application (Posma et al., 2020; See, 2024). Metformin may also cause gastrointestinal side effects including abdominal distension, diarrhea, cramps, nausea, and vomiting (Dixon et al., 2023). In addition, metformin aggravates renal toxicity by promoting the neutrophil extracellular trap (NETosis) response and enhancing renal ferroptosis (Cai et al., 2023). During the treatment of acute diseases, metformin may increase the risk of AKI, lead to a decline in renal function, and may also increase the risk of complications and death.

**Resveratrol.** Resveratrol, as a polyphenolic compound, is able to reverse the decreased expression of SIRT1 and the increased expression of the expression of junctional adhesion molecule-like (JAML), sterol regulatory element binding protein-1 (SREBP-1) and carbohydrate-response element (ChREBP) in DKD patients and HFD-induced DKD mice. Specifically, resveratrol regulates the JAML/SIRT1 lipid synthesis pathway through elevating the SIRT1 expression to reduce the expressions of SREBP-1 and ChREBP protein, further mitigating lipid deposition mediated by SREBP-1 and ChREBP, improving renal pathology and renal function in DKD patients and HFD-induced DKD mice (Gu et al., 2022).

Resveratrol attenuates mononuclear cell infiltration, smooth muscular cell migration, renal vessel sclerosis, and tubulointerstitial fibrosis induced by inflammation and oxidative stress after renal transplantation by regulating the SIRT1/FoxO1 signaling pathway to improve renal inflammation and oxidative stress injury. Resveratrol significantly increases the expression of SIRT1, reverses the elevated Ac-NF-κB p65 levels in kidney transplanted rats, and elevates glutathione (GSH), superoxide dismutase (SOD), and catalase (CAT) levels, while decreasing the levels of malondialdehyde (MDA) and iNOS. In addition, it inhibits the expression of proinflammatory cytokines and chemokines, including TNF-α, CD68, and IL-6 in kidney transplanted rats (Zhou et al., 2022). By increasing the expression of SIRT1, resveratrol can alleviate renal fibrosis and muscle atrophy in 5/6-nephrectomized (5/6Nx) rats. Resveratrol treatment effectively enhances the interaction between SIRT1 and FoxO1 in the muscle of 5/6Nx rats. This mechanism inhibits the acetylation of FoxO1, reduces the levels of TGF-β, connective tissue growth factor (CTGF), and the expression of fibrotic markers collagen I and fibronectin, alleviating the degree of fibrosis. In addition, resveratrol increases dystrophin expression, downregulates the expression of muscle atrophy-associated proteins, including atrogin-1, MuRF-1, and myostatin through enhancing the interaction between SIRT1 and FoxO1, thereby alleviating muscle atrophy. Notably, the renoprotective effects of resveratrol depend on the dose. The small dose (≥25 mg/kg) of resveratrol improves renal function, while high doses (≥50 mg/kg) aggravate renal fibrosis (Wang R. et al., 2022). In the hyperoxia-induced renal injury model, the use of resveratrol can improve glomerular, thylakoid stromal hyperplasia, swelling metaplasia of tubular epithelial cells, and inflammatory cell infiltration induced by hyperoxia exposure. The intervention with resveratrol increases the protein levels of SIRT1 and PGC-1α, Nrf1, Nrf2, and mitochondrial TFAM to activate the SIRT1-PGC-1α-Nrf1/2-TFAM signaling pathway, promote mitochondrial biogenesis, and effectively protect neonatal rats from hyperoxia-induced kidney damage. Additionally, resveratrol effectively reduces apoptosis by regulating the pathway (Shen et al., 2025).

Notably, resveratrol may aggravate renal injury in the case of renal ischemia–reperfusion. Resveratrol can aggravate H_2_O_2_-induced HK-2 cell injury by upregulating the expression of PTEN and inhibiting the phosphorylation of Akt. This indicates that resveratrol may exacerbate renal injury in the case of renal ischemia–reperfusion injury (Luo et al., 2025).

**Quercetin.** Quercetin, a plant-derived flavonol compound, possesses a variety of biological activities including antioxidant and anti-inflammatory, and is commonly found in nature and everyday food, and its powerful oxidative and biological functions are essential for human health (Qi et al., 2022b). Quercetin has a protective effect against kidney injury of the LPS-induced SAKI mice model and HK-2 cells treated with LPS. Quercetin pretreatment significantly inhibits LPS-induced apoptosis and inflammation to alleviate RTEC injury, including brush border loss, vacuolization, inflammatory cell infiltration, and necrosis by upregulating SIRT1 expression, inhibiting the NF-κB activity and reducing the expression of related pro-inflammatory factors (the level of IL-6, IL-1 and TNF-α) and pro-apoptotic protein (cleaved caspase-3, Bax and cleaved caspase-9) (Lu et al., 2021). The quercetin treatment mitigates renal tubule dilatation and kidney injury with intraepithelial vacuolar degeneration and interstitial inflammation of contrast agent-induced AKI (CI-AKI) diabetes mellitus mice. Mechanistically, quercetin significantly increases the expression of SIRT1 and reduces oxidative stress by increasing Nrf2/HO-1/SOD1. Quercetin also inhibits the expression of pro-apoptotic proteins (Bax and cleaved caspase-3) while increasing the Bcl-2 protein level to mitigate cell apoptosis induced by contrast agent. In addition, quercetin inhibits the polarization of M1 macrophages in a CI-AKI mice model to exert anti-inflammatory effects (Wu Z. et al., 2024).

Quercetin, a key active constituent in the Jiangya Tongluo decoction (JYTL) for treating HTN, works in concert with tanshinone IIA and adenosine to exert renoprotective and anti-fibrotic effects to alleviate pathological changes including mitochondrial swelling, glomerular ischemia and sclerosis, interstitial fibrosis, inflammatory cell infiltration, and compensatory dilation of renal tubules in HTN mice. JYTL enhances mitochondrial function; boosts the expressions of SIRT1, PGC-1α, Nrf1, and TFAM; and activates the PINK1/Parkin-mediated mitophagy pathway to reduce renal injury in HTN mice. Additionally, JYTL can ameliorate renal function by inhibiting ROS production and modulating mitochondrial dynamics in spontaneous hypertensive rats, thereby improving mitochondrial functionality to exert renoprotective effects (Zhao et al., 2024).

Quercetin alleviates the development of renal fibrosis in an UUO-induced renal fibrosis mice model and NRK-52E cells treated with Ang II by activating the mitochondrial autophagy mediated by the SIRT1/PINK1/Parkin pathway. Specifically, quercetin significantly increases the expression of SIRT1, enhances the expression of mitochondrial autophagy signal-related proteins LC3II, PINK1 and Parkin, while reducing the level of p62 to enhance mitochondrial autophagy. Quercetin pretreatment reduces the production of mitochondrial ROS by enhancing mitochondrial autophagy, improving the reduction of mitochondrial membrane potential and alleviating mitochondrial dysfunction, thereby alleviating the aging of RTESs to exert anti-renal fibrosis effect (Liu T. et al., 2020).

The protective effect of quercetin against renal injury in CdCl-induced rats are induced mainly by increasing the expression and nuclear activity of SIRT1 and inhibiting the SIRT1-dependent deacetylation of X-box binding protein 1 spliced (Xbp-1s) and eukaryotic Initiation Factor 2 alpha (eIF2α) to ERS. Additionally, quercetin increases the protein expression of Bax, cleaved caspase-3, Bax/Bcl-2 ratio, and cleaved caspase-3, thereby significantly inhibiting cell apoptosis (Alshammari et al., 2021).

*In vitro* experimental, quercetin inhibits the growth and function of normal thyroid cells and interferes with the ability of thyroid hormone metabolism. These effects have also been confirmed *in vivo* using rodent models (Giuliani et al., 2024).

**Curcumin.** Curcumin, the main constituent of *Curcuma longa* rhizomes, boasts a variety of pharmacological effects, including the anti-inflammatory, immunomodulatory, antioxidant, and lipid-modulating properties (Sadeghi et al., 2023; Yan et al., 2025). By activating the SIRT1/Nrf2/HO-1 pathway, curcumin mitigates pathological changes including RTEC swelling, vacuolar degeneration, cell shedding, and interstitial inflammatory cell infiltration in the aristolochic acid (AA-I)-induced renal injury mice model and the NRK-52E cells treated with AA-I. The AA-I exposure notably elevates the levels of pro-apoptotic protein (Bax, caspase-9, cleaved caspase-9, caspase-3, and cleaved caspase-3), while reducing the expression of anti-apoptotic and anti-inflammatory proteins (Bcl-2, SIRT1, Nrf2, NQO1, HO-1, and Keap1). These molecular changes are counteracted by the administration of curcumin (Liu Z. et al., 2022). Tetra hydro curcumin (THC) exhibits more pronounced physiological and pharmacological activities than curcumin, including stronger anti-inflammatory and antioxidant effects. In the CLP-induced SAKI mice model, the treatment of THC can upregulate the expression and activity of SIRT1 and downregulate the expressions of ac-p65 and ac-FoxO1 and improve renal function and renal tissue injury in SAKI mice. Additionally, THC increases the activity of endogenous antioxidant enzymes (SOD, GSH, CAT, and GPX) and reduces the protein and mRNA expressions of proinflammatory factor (IL-1β, IL-6, and TNF-α) to inhibit oxidative stress, inflammatory responses, and prevent cell apoptosis, thereby maintaining renal function and alleviate SAKI-induced by CLP (Li L. et al., 2021).

**Melatonin (N-acetyl-5-methoxytryptamine; MLT).** The combined therapy of melatonin and ascorbic acid restores the SIRT1 levels and alleviates the glomerular damage, the dilation of renal tubule lumen, and tissue congestion in the CLP-induced SAKI model. Melatonin reduces oxidative DNA damage and NF-κB levels and effectively decreases the markers of inflammation (TNF-α, IL-1β, and IL-6) and DNA damage (8-hydroxy-2′-deoxyguanosine). This combined treatment demonstrates a significant synergistic effect in combating inflammation, cell damage, and oxidative stress induced by sepsis (Üstündağ et al., 2023).

Melatonin can alleviate glomerular hypertrophy and excessive accumulation of mesangial matrix, delay the progression of DKD, and improve renal dysfunction by activating the AMPK/SIRT1 pathway in both the STZ-induced DKD model and the NEK-52E cell treated with HG. Specifically, melatonin increases the expressions of SIRT1 and FoxO3a (the downstream molecule of SIRT1), reducing MDA expression and increasing the expression of GSH and GPX to ameliorate oxidative stress. In addition, melatonin also increases the expression of liver kinase B1 (LKB1) in renal tissue and elevates the level of p-AMPK in the STZ-induced DKD model and the NEK-52E cell treated with HG. Melatonin treatment enhances the nuclear translocation of Nrf2 in NRK-52E cells under HG conditions and improves Nrf2-mediated antioxidant ability. Notably, melatonin mediates the nuclear output of PGC1α by upregulating the expression of SIRT1 and increases the expression of mitochondrial biogenesis markers PGC1 α and TFAM after melatonin treatment, thereby enhancing autophagy and mitochondrial health (Siddhi et al., 2022).

**SRT1720.** The expression of SIRT1 and angiotensin-converting enzyme 2 (ACE2) is reduced in the HG-induced diabetes rat model and NRK-52E cells treated with HG. However, SRT1720 activates SIRT1 expression and reverses the increased A disintegrin and metalloproteinase (ADAM) 17 and the decreased tissue inhibitor of metalloproteinase 3 (TIMP3, an inhibitor of ADAM 17) to reverse the imbalance of renal TIMP3/ADAM17 induced by HG, therefore upregulating the ACE2 expression in HG-treated NRK-52E cells, promoting the translation of Ang II to Ang (1–7), and playing a renoprotective role in DKD (Guo Z. et al., 2024).

SRT1720 significantly increases the expression of SIRT1; alleviates oxidative stress, renal fibrosis, and apoptosis; and effectively reduces interstitial fibrosis and inflammatory cell infiltration in a UUO-induced renal fibrosis mice model. Mechanistically, SRT1720 reduces oxidative stress by decreasing the levels of MDA, TGF-β1/connective tissue growth factor (CTGF), and glutathione (GSH), increasing the levels of SOD, GPX, thereby protecting cells from ROS-induced damage and playing a protective role against UUO-induced tubulointerstitial fibrosis (Ren et al., 2017).

In a trichloroethylene (TCE) sensitized mouse model, the expression of SIRT1 protein and heat shock protein 70 (HSP70) is significantly reduced in the renal tubules, while the levels of HSP70 in serum and urine significantly increased, and the content of inflammatory cytokines is significantly increased. However, these abnormal changes are reversed after treatment with SRT1720. HSP70, as a ligand for TLR4, can interact with TLR4 to jointly trigger an inflammatory response, whereas the activation of SIRT1 effectively inhibits this process. Therefore, SRT1720 reduces the release of HSP70 by enhancing the expression of SIRT1, thereby reducing the interaction between HSP70 and TLR4 and subsequently alleviating immune damage in RTECs (Zhang X. et al., 2022). Additionally, the increase of TNF-α induced by TCE sensitivities in renal vascular endothelial cells inhibits the expression of SIRT1, thereby inhibiting the PINK1-Parkin mitochondrial autophagy pathway, leading to mitochondrial dysfunction and inducing apoptosis of renal vascular cells. Notably, SRT1720 significantly restores mitochondrial function in renal endothelial cells and enhances mitochondrial autophagy, improving glomerular mesangial hyperplasia and renal tubular injury by regulating SIRT1 expression. Consequently, SRT1720 intervenes in the mitochondrial autophagy and apoptosis of renal vascular endothelial cells in immune-mediated renal injury induced by TCE sensitization (Xie H. et al., 2024).

**SRT2104.** SRT2104, a SIRT1 agonist, is currently less utilized in the treatment of kidney diseases. However, SRT2104 can effectively reduce the expansion of mesangial matrix and accumulation of fibrosis to alleviate diabetic complications by regulating the SIRT1/p53/Nrf2 signaling pathway in an STZ-induced diabetes mice model. Specifically, SRT2104 can enhance the expression and activity of SIRT1 in the kidneys, promote the deacetylation of p53, and activate the antioxidant signaling pathway of Nrf2, thereby providing significant protection against diabetic-induced kidney oxidative stress, inflammatory response, fibrosis, and glomerular structural remodeling (Ma et al., 2019; Chang et al., 2024).

In cadmium-induced renal injury mice model and the NRK-52E cells treated with cadmium, SRT2104 can reduce the increased acetylation level of FoxO1 caused by cadmium through up-regulating SIRT1 expression and activity to mitigate the nucleus shrinkage, chromatin condensation and lumen is irregular, improving pathological injury of the kidney. Specifically, SRT2104 enhances the expression and activity of SIRT1, the deacetylation level of FoxO1 and the transcription level of Ras-associated protein 7 (Rab7) suppressed by cadmium to restore the fusion of autophagosomes and lysosomes blocked by cadmium, and alleviate cadmium-induced autophagy inhibition, thereby intervening in cadmium-induced kidney damage (Ruan Y. et al., 2024).

##### 4.1.1.2 The activator of SIRT3

**Honokiol.** Honokiol, the predominant biphenolic constituent extracted from the magnolia tree, exhibits a wide range of biological activities including antioxidant, anti-inflammatory, and anti-fibrotic effects (Chen C. et al., 2021). Honokiol reduces the focal necrosis and apoptosis in proximal tubules in the cisplatin-induced AKI mice model and HK-2 exposed to cisplatin by restoring SIRT3 expression and enhancing AMPK activity in RTECs. Additionally, honokiol preserves the phosphorylation of dynamin-related protein 1 (Drp1) at the Ser637 site and blocks its translocation to the mitochondria, which in turn prevents mitochondrial fragmentation and subsequent cell damage and death to mitigate renal injury induced by cisplatin (Mao et al., 2022). Additionally, honokiol can reduce fatty acid oxidation (FAO), fatty acid deposition, and improve kidney function by activating SIRT3 in cisplatin-induced AKI mice model and RTECs treated with cisplatin. At the mechanistic level, honokiol inhibits the acetylation of LKB1 and upregulates the phosphorylation of AMPKα and acetyl-CoA carboxy (ACC) by activating SIRT3, improving FAO. Additionally, after honokiol activates SIRT3, it increases ATP production and reduces the levels of ROS to improve mitochondrial function (Li et al., 2020).

The SIRT3 knockout mice are susceptible to hyperacetylated mitochondrial proteins and severe renal fibrosis, whereas honokiol restores the SIRT3 expression to alleviate fibrosis in a UUO-induced renal fibrosis mice model. The antifibrotic effect of honokiol is achieved by upregulating the activity and expression of SIRT3, deacetylating pyruvate dehydrogenase E1α (PDHE1α) at lysine 385, further inhibiting the PDHE1α phosphorylation and restoring pyruvate dehydrogenase complex (PDH) enzyme activity, thus preventing kidney fibrosis in the UUO-induced renal fibrosis mice model (Zhang et al., 2021b).

It is worth noting that improper dosage of honokiol may affect nephrotoxicity and exacerbate kidney damage. Research has shown that the high dose of the honokiol microemulsion (0.6 μg/mL) induces developmental toxicity in rats and zebrafish by inducing oxidative stress (Li H. et al., 2022).

**Melatonin (N-acetyl-5-methoxytryptamine; MLT).** Melatonin, a compound that helps regulate sleep and circadian rhythms, is currently widely used in populations with sleep disorders (Vasey et al., 2021). However, melatonin has a protective effect on the kidneys against injury (Chen L. et al., 2021). The melatonin treatment mitigates kidney injury in sepsis patients, CLP-induced SAKI mice model, and HK-2 cells treated with LPS. This effect depends on the activation of SIRT3. Mechanistically, melatonin increases the expression of mitochondrial markers (PINK1, PRKN, and LC3-II), enhances mitochondrial autophagic flux by upregulating the expression of SIRT3, and increasing the SIRT3-mediated TFAM deacetylation level at the K154 site to promote mitochondrial autophagy flux, thereby alleviating SAKI (Deng et al., 2024). In addition, melatonin inhibits ferroptosis and renal tubule dilation to improve renal injury in the CLP/LPS-induced AKI mice model by activating SIRT3 and up-regulating the Nrf2/HO-1 pathway to inhibit ferroptosis induced by SAKI (Qiu et al., 2022).

**Metformin.** Metformin can reduce the vacuolated PTECs, dilated tubules, collagen deposition, and mitochondrial morphological abnormalities to protect PTECs from the impact of FA/IRI by activating the AMPK pathway and promoting the SENP1-SIRT3 axis in FA/IRI-induced AKI mice model. Additionally, metformin reduces the acetylation of mitochondrial SOD2 by increasing the expression of SIRT3, decreases the production of ROS, and subsequently restores mitochondrial ATP levels to reverse mitochondrial morphological changes, mitigate apoptosis, inflammation, and fibrosis induced by FA/IRI (Zhu et al., 2023).

##### 4.1.1.3 The activator of SIRT6

**Daphnetin.** Daphnetin alleviates the dilation of proximal renal tubules, the formation of tubules, and the detachment and necrosis of RTECs to exerts renoprotective effects in the cisplatin-induced AKI murine model and HK-2 cells treated with cisplatin through upregulating SIRT1 and SIRT6 expressions. At the molecular level, the use of daphnetin increases the expressions of SIRT1 and SIRT6, while decreasing NADPH oxidase 4 (NOX4) levels, promoting Nrf2 nuclear translocation and enhancing the expression of downstream antioxidant enzymes, including HO-1 and NAD(P)H quinone oxidoreductase 1 (NQO1), thereby ameliorating oxidative stress and mitochondrial dysfunction. Additionally, daphnetin significantly suppresses the inflammatory pathways involving NF-κB, mitogen-activated protein kinase (MAPK), and the apoptotic pathways mediated by p53 and cleaved caspase-3 to alleviate inflammatory responses and apoptosis. These coordinated pharmacological actions collectively contribute to the marked attenuation of renal injury induced by cisplatin. Notably, daphnetin does not reduce the anti-tumor effects of cisplatin but instead enhances its therapeutic effect (Fan et al., 2020c).

**Isoorientin.** Isoorientin upregulates the expression of SIRT1 and SIRT6 to effectively reduce oxidative stress, inflammation, and prevent cell apoptosis in the cisplatin-induced AKI mice model and RTECs treated with cisplatin. Mechanically, isoorientin increases the SIRT1 and SIRT6 expressions, promotes Nrf2 translocation, and activates the expression level of antioxidant enzymes, including HO-1 and NQO1 to inhibit the expression levels of NOX4 and ROS production, thereby reducing oxidative stress, alleviating mitochondrial dysfunction, and improving kidney injury of cisplatin-induced AKI mice. Isoorientin treatment also significantly reduces the phosphorylation of NF-κB, high-mobility group box 1 (HMGB1), JNK, ERK, and p38; blocks the activation of NF-κB, HMGB1 and MAPK signaling pathways; and inhibits the inflammatory response. Additionally, isoorientin decreases the expressions of cleaved caspase-3, p-p53, and Bax, while increasing the expression of Bcl-2 to inhibit apoptosis (Fan et al., 2020b).

**Diosgenin.** Diosgenin alleviates mesangial matrix dilation and GBM thickening in the db/db mice and protects db/db mice from podocyte injury by increasing SIRT6 expression to reduce lipid accumulation. The reduction of lipid accumulation by upregulation of SIRT6 expression may be related to the distinct effects of pyruvate dehydrogenase kinase 4 (PDK4) and angiopoietin-like 4 (ANGPTL4) downstream of SIRT6. However, the mechanism of how SIRT6 affects PDK4 and ANGPTL4 to reduce lipid accumulation remains unclear (Wang Z. et al., 2022).

### 4.2 Inhibitors

HDACs play a crucial role in epigenetic regulation, mediating the deacetylation process of both histones and non-histone proteins (Liu, 2021). Over the past decades, studies have shown that HDAC inhibitors can prevent and possibly treat various types of AKI (Hyndman, 2020). In addition, changes in the expression of HDACs have been observed in kidney samples from patients and in an animal model of various kidney diseases. The administration of HDAC inhibitors has exerted protective effects on the kidneys both in *in vitro* and *in vivo* studies (Zhang and Cao, 2022a). HDAC inhibitors are organic compounds that inhibit histone deacetylation, exerting protective effects on the kidney by suppressing inflammatory responses and regulating cellular processes, including apoptosis and autophagy. These inhibitors directly act on HDACs and can also indirectly affect the activity of HATs, which modulate the level of acetylation and deacetylation in the cell nucleus. HDACs lead to chromatin condensation (chromatin blocking state) by removing acetyl groups from histone lysine residues, ultimately leading to transcriptional repression (Wei Y. et al., 2022). Most of the inhibitors with renal protection effects target HDAC1-11 and mediate related pathways to play a renal protection role. In addition, inhibitors that inhibit the expression and/or activity of SIRT1, SIRT3, and SIRT7 have also been shown to have renal protective effects. Therefore, we also discuss the mechanism of these inhibitors targeting SIRT1, SIRT3, and SIRT7 (Table 4).

**TABLE 4:** The mechanism of SIRT inhibitors and HDACs inhibitors in renal disease.

Enzymes	Inhibitor	Diseases	Pathway	Pathophysiological	References
SIRTs					
SIRT1	Metformin	DKD	MiR-34a/SIRT1	Apoptosis	Zhuang et al. (2024)
SITR2	AGK-2	AKI	SIRT2/FoxO3a/FASL	Apoptosis	Wang et al. (2017)
			SIRT2/KIM-1/NGAL FoxO1/TNF-α/IL-1β/IL-6	Autophagy and inflammation	Yu et al. (2025)
	Tenovin-1	DKD	SIRT2/STAT3/EGFR/PDGFR/TGF-β/	Fibrosis, apoptosis, and inflammation	Kundu et al. (2022)
SITR7	Zn^+^/94971	AKI	SIRT7/ROS/NLRP3	Pyroptosis	Guo et al. (2024a)
HDAC					
HDAC1	Trichostain A	PKD	HDAC1/Cux1/GRG4/p27	Cell proliferation and cyst growth	Livingston et al. (2019)
	Valproic acid	AKI	HDAC1/H3K27/GPX4/ACSL4	Ferroptosis	Wei et al. (2022a)
	31c	AKI	HDAC1/PHD2/NGAL	Renal tubule injury and cell necrosis	Wei et al. (2022a)
	Rutin	DKD	PI3K/AKT/Mtor/HDAC1/CD31	Fibrosis and autophagy	Dong et al. (2023)
HDAC2	Trichostain A	Renal fibrosis	HDAC2/CSF-1/α-SMA	Fibrosis and inflammation	Marumo et al. (2010)
	Valproic acid	AKI	HDAC2/BMP-7/KIM-1/Bax/Bcl-2	Apoptosis	Ma et al. (2017)
	Sulforaphane	Renal fibrosis	HDAC2/BMP-7/Smad1/5/8/TGF-β	Fibrosis	Kong et al. (2021)
HDAC3	RGFP966	Renal fibrosis	HDAC3/NCoR/NF-κB/Klotho/	Fibrosis	Chen et al. (2021b)
			HDAC3/NF-κB p65	Fibrosis	Wang et al. (2024b)
	Astragaloside I	DKD	HDAC3/Klotho/TGF-β1/Smd2/3	Fibrosis	Zhang et al. (2024b)
HDAC4	TMP195	AKI	HDAC4/H4K14/NGAL/KIM-1	Apoptosis and inflammation	Zhang et al. (2020a)
	TMP269	AKI	HDAC4/caspase-3/Bax/p53	Apoptosis, autophagy, and inflammation	Li et al. (2022b)
HDAC5	Trichostain A	Diabetes	HDAC5/TGF-β/α-SMA	Fibrosis	Xu et al. (2021b)
HDAC6	Trichostain A	DKD	HDAC6/TFEB	Fibrosis	Li et al. (2024b)
	F7	AKI	HDAC6//ERK1/2/p38/NF-κB	Inflammation	Liu et al. (2019)
	23BB	AKI	HDAC6/p-PERK/EIF2α	Endoplasmic stress, inflammation, and apoptosis	Hao et al. (2020)
	CAY10603	DKD	HDAC6/NLRP3/caspase1	Macrophages pyroptosis, and fibrosis	Hou et al. (2022)
	Tubacin2	DKD	HDAC6/P62/LC3-II/Beclin-1	Autophagy	Liang et al. (2020)
		PKD	HDAC6/cAMP	Cell proliferation and cyst growth	Cebotaru et al. (2016)
	Compound 26	PKD	HDAC6/α-tublin	Cell proliferation and cyst growth	Cao et al. (2024)
HDAC8	PCI-34051	AKI	HDAC8/NGAL/p53/p21/CDK2	DNA damage and repair, apoptosis	Wang et al. (2024c)
		Renal fibrosis	HDAC8/TGF-β/Smad/STAT3/β-catenin	Fibrosis	Zhang et al. (2020b)
HDAC9	TMP-195	Renal fibrosis	HDAC9/STAT1	Renal tubulointerstitial fibrosis	Zhang et al. (2023c)
HDAC11	Quisinostat	Renal fibrosis	HDAC11/AP-2α/KLF15	Fibrosis and inflammation	Mao et al. (2020)

#### 4.2.1 SIRT inhibitors

##### 4.2.1.1 The inhibitors of SIRT1

**Metformin.** In STZ-induced diabetic mice and HG-treated podocytes, the expression of miR-34a is significantly downregulated and the expression of SIRT1 is upregulated, accompanied by glomerular mesangial hypertrophy and apoptosis. Metformin elevates the miR-34a levels and decreases SIRT1 levels by directly targeting SIRT1 3 ′-UTR to recover podocyte viability and inhibit podocyte apoptosis (Zhuang et al., 2024).

**EX-527.** In the HFD-induced diabetic mouse model, the expression of SIRT1 is upregulated, but the expression of SIRT3 is downregulated. In addition, pathological changes including glomerular sclerosis, tubular dilation, interstitial nodular sclerosis, and fibrous hyperplasia are observed in this mouse model. EX-527 reduces these pathological changes by inhibiting SIRT1 and increasing SIRT3 expression. Mechanistically, EX-527 treatment can significantly reduce the levels of pro-inflammatory factors TGF-β1, IL-1β, and IL-6 to mitigate the inflammatory response. EX-527 enhances the antioxidant activity of kidney tissue by restoring the activity of SOD and reducing the expressions of MDA and ROS to alleviate oxidative damage. In addition, EX-527 inhibits abnormal expansion of the extracellular mesangial matrix, reduces the occurrence of glomerular sclerosis by reducing EGFR and platelet-derived growth factor receptor (PDGFR) phosphorylation levels, and blocks pro-fibrotic signaling pathways, further downregulating the expressions of fibrosis markers α-SMA, collagen-1, TGF-β, vimentin, α-tubulin, and fibronectin (Kundu et al., 2020).

##### 4.2.1.2 The inhibitors of SIRT2

**AGK-2.** AGK2, as a potent and highly selective SIRT2 inhibitor, can alleviate apoptosis in the RIR mouse model by decreasing the SIRT2 expression. Specifically, AGK-2 significantly inhibits the SIRT2 expression and the interaction of SIRT2 with FoxO3a to reduce the deacetylation of FoxO3a and nuclear translocation and decreases the Fas Ligand (FasL) expression and the activation of caspase-8 and caspase-3 (Wang et al., 2017). The pathological changes including RTEC detachment, tubular dilatation, and epithelial flattening are observed in CLP-induced SAKI and HK-2 cells treated with LPS. Notably, the expression of SIRT2 is upregulated within 12 h after CLP processing. AGK-2 can inhibit the expressions of SIRT2 to alleviate these pathological changes and exert renal protective effects. Mechanically, AGK2 reduces the expression of renal function and injury markers KIM-1 and NGAL mRNA, thus mitigating renal tubular injury and improve renal functions. Additionally, AGK2 significantly inhibits SIRT2 activity, further reducing the deacetylation levels of FoxO1 at K262, K265, and K274 sites to enhance autophagy in RTECs. The anti-inflammatory effect of AGK2 is manifested in reducing the expressions of IL-6, TNF-α, and IL-1β in renal tissue induced by CLP, thereby alleviating inflammatory response and elevating the survival rate of SAKI model mice (Yu et al., 2025).

**Tenovin-1.** Tenovin-1 is a potent and selective SIRT1 and SIRT2 inhibitor, which can significantly inhibit the enlargement and vacuolation of proximal and distal tubes, thickening of GBM, and lipid droplet accumulation in the HFD-induced DKD rat model and NRK-52E cells treated with HG. Mechanically, tenovin-1 reverses the increased claudin-1, SIRT1, and SIRT2 expressions induced by HFD and inhibits the phosphorylation of STAT3, EGFR, and PDGFR. Tenovin-1 reduces the expressions of E-cadherin, α-SMA, TGF-β, and collagen-1 protein and the level of ECM in the kidneys, further alleviating renal fibrosis and reducing HFD-induced renal apoptosis. In addition, the use of tenovin-1 inhibits the expression levels of pro-inflammatory cytokines IL-1β, IL-6, and the 8-OHDG to reduce the inflammatory response and the oxidative damage of lipids or DNA. Notably, tenovin-1 increases the expressions of SIRT3 and SIRT4 (Kundu et al., 2022).

**Berberine.** Berberine reduces collagen deposition and renal tubular necrosis and cystic dilation in a cisplatin-induced renal injury rat model by inhibiting SIRT2. Mechanistically, berberine inhibits the expression of murine double-minute 2 (MDM2) and SIRT2, downregulates the purinergic P2X7 receptor (P2X7R) and p-ERK1/2 expression, and increases the expression of dual specificity phosphatase 6 (DUSP6) to effectively mitigate cisplatin-induced renal histopathological damage and improve renal function. Additionally, it downregulates fibrosis markers (galectin-3 and α-SMA), thus significantly attenuating fibrotic progression (Ahmedy et al., 2022).

##### 4.2.1.3 The inhibitors of SIRT7

**Zn^+^/94971.** In the CLP-induced SAKI rat model and LPS-stimulated HK-2 cells, Zn^+^/94971 reduces renal tubular injury and apoptosis by inhibiting SIRT7 expression. Mechanistically, Zn^+^/94971 reduces parkin deacetylation by inhibiting SIRT7 expression and then activates mitochondrial autophagy, thereby reducing ROS accumulation, blocking the activity of NLRP3 inflammasome mediated by ROS to reduce the pyroptosis of RTECs, and ultimately improve the kidney injury caused by sepsis (Guo J. et al., 2024).

#### 4.2.2 HDAC inhibitors

##### 4.2.2.1 The inhibitors of HDAC1

**Trichostatin A (TSA).** A growing body of evidence suggests that phenotypic changes in macrophages play a critical role in the development and progression of CKD (Chung et al., 2023). The phenotypes of macrophages are mainly divided into two major categories: one is the M1 macrophages that promote inflammation, and the other is M2 macrophages that have anti-inflammatory and repair properties. M2 macrophages can be further subdivided into M2a and M2c subtypes. However, the overactivation of M1 macrophages and certain M2 macrophages that promote fibrosis may facilitate the process of fibrosis. M2 macrophages may play a dual role in regulating kidney fibrosis (Liu T. et al., 2021; Luo et al., 2023b). TSA, as a pan-inhibitor targeting multiple HDACs, can alleviate inflammatory responses and renal fibrosis by inhibiting HDACs and downregulating the expression of M2a macrophages in the UUO-induced renal fibrosis mice model and NRK-52E cells. By inhibiting the expression of HDACs, TSA promotes the transformation of pro-inflammatory M1 macrophages into anti-inflammatory M2 macrophages and reduces the infiltration of M2a macrophages. At the same time, TSA can increase the number of anti-inflammatory M2c macrophages, thereby inhibiting the inflammatory response, myofibroblast activation, and ECM deposition. TSA also decreases the expressions of α-SMA and fibronectin induced by TGF-β1 in RTECs and fibroblasts, further alleviating renal fibrosis in the UUO model (Tseng et al., 2020).

TSA delays cyst growth in the ADPKD mice model with Pkd1 deletion by inhibiting HDAC1 and HDAC3. Specifically, TSA alleviates the inhibition of cyclin kinase inhibitors p27 by cut-like homeobox 1 (Cux1) and groucho-related gene 4 (Grg4) and increases the expression of p27 by inhibiting the elevated expressions of HDAC1 and HDAC3 in the kidneys of Pkd1-deficient mice to inhibit cell proliferation in the nephrogenic zone, ultimately reducing cysts in neonatal Pkd1-deficient mice (Livingston et al., 2019).

**Valproic acid (VPA).** VPA can reduce pathological changes including RTEC death, cell shedding, and interstitial edema in AKI patients, cisplatin-induced AKI mice model, and cisplatin-stimulated HK-2 cells by inhibiting HDAC1 and HDAC2 activities. Specifically, VPA treatment is able to inhibit the expressions of HDAC1 and HDAC2 while increasing the levels of H3K27Ac. This action further upregulates the GPX4 expression and inhibits long-chain family member 4 (ACSL4) expression, thereby reducing cisplatin-induced ferroptosis in cisplatin-induced AKI mice (Li Y. et al., 2023).

In the ADPKD model with Pkd1 mutations, the inhibition of Class I HDACs (primarily HDAC1) with VPA suppresses the body curvature phenotype, declines the rate of cyst growth, and improves renal function (Cao et al., 2009). However, the mechanism by which VPA’s inhibitory effect on HDAC1 alleviates PKD remains unclear.

The use of VPA during pregnancy may have adverse effects on the fetus. Studies have shown that prenatal exposure to valproic acid (VPA) is associated with an increased risk of autism spectrum disorder (ASD) in offspring, especially for those exposed to VPA in the early stages of pregnancy (the first 3 months), whose risk of ASD is significantly enhanced (Nicolini and Fahnestock, 2018; Taleb et al., 2021). In addition, VPA may induce encephalopathy, mainly manifested as acute or subacute encephalopathy, and is associated with hyperammonemia, L-carnitine deficiency and urea, circulating enzyme dysfunction (Wu et al., 2021).

**Hybrid inhibitor-31c.** The hybrid inhibitor-31c is a hybrid inhibitor targeting hypoxia-inducible factor prolyl hydroxylase 2 (HIF-PHD2) and HDAC1/2/6. The 31c can significantly inhibit the expressions of PHD2 and HDAC1/2/6 to alleviate the widening of proximal renal tubules, the local necrosis of epithelial cells, prevent the progression of AKI, and improve renal dysfunction in the cisplatin-induced AKI mouse model and HK-2 cells treated with cisplatin. At the molecular level, 31c downregulates the expression of NGAL (the marker of renal tubular injury), reduces the plasma BUN/SCr levels, and increases the level of erythropoietin (EPO). Notably, 31c does not affect the antitumor effects of cisplatin in cancer cells while effectively protecting HK-2 cells from cisplatin-induced toxicity (Wei H. et al., 2022).

**7-Hydroxycoumarin (7-HC).** 7-Hydroxycoumarin (7-HC) can alleviate renal tubule expansion, epithelial cell detachment, and necrosis in the colistin-induced kidney injury mice model and the mice RTECs treated with colistin, and this protective effect is achieved by reducing the level of HDAC1, activating the Nrf2 signaling pathway to upregulate the protein expressions of Nrf2, HO-1, and H3K27AC, thereby enhancing the kidney’s antioxidant capacity. 7-HC also reduces the levels of ROS and MDA and increases the activities of SOD and CAT to alleviate colistin-induced cell damage. Additionally, 7-HC treatment significantly inhibits the increased activities of caspase-3 and caspase-9 to alleviate apoptosis (Wang et al., 2020).

**Rutin.** Rutin can reduce renal tubule dilation, glomerular hypertrophy, and collagen deposition by inhibiting the PI3K/Akt/mTOR signaling pathway to decrease the HDAC1 activity in DKD db/db mice and HG-treated glomerular endothelial cells (GEnCs). Based on this, rutin can upregulate the cell adhesion molecule-1 (CD31) and vascular endothelial-cadherin (VE-cadherin) expression levels, while downregulating the α-SMA and collagen 1 expression levels, alleviating EMT through elevating LC3B II/LC3B I ratio and beclin 1 expression and restoring autophagy in db/db mice and HG-treated GEnC (Dong et al., 2023).

##### 4.2.2.2 The inhibitors of HDAC2

**Trichostatin A.** In UUO-induced renal tubulointerstitial injury model and NRK-52E cells stimulated by TNF-α, TSA treatment alleviates interstitial fibrosis and inflammatory responses by reversing the upregulated HDAC1 and HDAC2 expressions induced to inhibit colony-stimulating factor-1 (CSF-1, A cytokine that mediates macrophage infiltration in the early stages of UUO), further reducing inflammation. Additionally, TSA reduces fibrosis marker α-SMA and collagen 1α expression to mitigate the renal interstitial fibrosis induced by UUO (Marumo et al., 2010).

**Valproic acid (VPA).** VPA inhibits HDAC2 expression and promotes the level of bone morphogenetic protein-7 (BMP-7) to reduce the necrosis of renal tubules, tubular formation, and apoptosis in the cisplatin-induced AKI rat model and HK-2/mTEC cells treated with cisplatin. Mechanically, VPA effectively reduces the protein levels of renal injury marker KIM-1 and Bax, increases Bcl-2 expression, and inhibits the activity of caspase-3, therefore alleviating the kidney injury and apoptosis of RTECs induced by cisplatin. This series of processes are based on the effect of VPA in inhibiting HDAC2 activity and increasing BMP-7 expression (Ma et al., 2017).

**Sulforaphane.** Sulforaphane significantly inhibits HDAC2 activity to alleviate ECM, collagen deposition, glomerular enlargement, and mesangial matrix enlargement in the STZ-induced renal fibrosis mice model and HK-11 cells treated with HG/palmitate. Specifically, sulforaphane increases the BMP-7 expression through inhibiting the activating of HDAC2, activates Smad1/5/8, and suppresses the TGF-β/Smad pathway, thereby mitigating renal fibrosis. However, the overexpression of HDAC2 reduces the expression of BMP-7 and eliminates the protective effect of SFN against STZ/HG/palmitate-induced fibrosis (Kong et al., 2021).

**α-cyperone (α-CYP).** The α-cyperone (α-CYP) intervention significantly inhibits the abnormal upregulation of HDAC2 expression and reduces the changes including the necrosis, degeneration of renal tubules, interstitial fibrosis, and neutrophil infiltration in the STZ-induced diabetic rat model with I/R treatment and HG-treated NRK-52E cells. The α-CYP pretreatment prevents the overexpressions of MDA and MPO (myeloperoxidase, the marker of neutrophil infiltration) induced by I/R, thereby protecting the kidneys from oxidative stresses and inflammatory mediators. Additionally, α-CYP significantly reduces the expression of fibrotic markers hydroxyproline and relative pro-fibrotic factors to alleviate renal fibrosis (Daude et al., 2024).

##### 4.2.2.3 The inhibitors of HDAC3

**RGFP966.** The RGFP966 selectively inhibits HDAC3 and alleviates renal tubular injury and fibrotic lesions including renal tubular atrophy and progressive interstitial fibrosis in the UUO/AAN-induced renal fibrosis mice model and the HK-2/HEK-293 cells stimulated by TGF-β. Mechanically, RGFP966 reduces the inhibitory effect of HDAC3 on Klotho transcription by forming an inhibitory complex with NCoR/NF-κB and effectively alleviates the abnormal expression of α-SMA, collagen-1, E-cadherin, and BMP-7 in UUO and AAN mice (Chen F. et al., 2021).

RGFP966 increases the acetylation level of NF-κB p65 at Lys122 by inhibiting HDAC3 activity to reduce collagen deposition in a UUO/IRI-induced renal fibrosis mouse model. This acetylation directly blocks NF-κB p65 binding to DNA and inhibits p65 interaction with kB-DNA by increasing steric hindrance. In addition, RGFP966 downregulates the expressions of collagen I, fibronectin, and α-SMA, thereby inhibiting IRI-induced fibroblast activation, ECM production, and deposition. Together, these effects reduce the degree of fibrosis induced by UUO/IRI (Wang et al., 2024b).

**Trichostatin A (TSA).** TSA mitigates kidney pathological changes including focal tubular atrophy and interstitial fibrosis and decreases the Klotho expression in adenine-induced CKD mouse model. This effect is mainly achieved by inhibiting HDAC3 expression to enhance the peroxisome proliferator-activated receptor γ (PPARγ, a key nuclear transcription factor) acetylation on K240 and K265, promote the binding of PPARγ and Klotho promoter, and increase the transcription of Klotho and thus upregulation of Klotho (Lin et al., 2017).

**Astragaloside I/IV.** Astragaloside I is a bioactive saponin extracted from *Astragalus membranaceus*. Astragaloside I can significantly mitigate pathological changes including tubule dilation, glomerular hypertrophy, and the GBM thickening and mesangial matrix in db/db mice and HG-treated SV40-MES-13 cells. Mechanically, astragaloside I directly binds to HDAC3, downregulates the expression of HDAC3, while upregulates the expression of Klotho to block the activation of the TGF-β1/Smad2/3 signaling pathway through synergistic inhibition of HDAC3 and TGF-β1, thereby mitigating the renal fibrosis process of DKD (Zhang X. et al., 2024). In addition, astragaloside IV inhibits apoptosis of RTECs and impedes the progression of DKD by reducing oxidative stress and inflammation and regulating key signaling pathways, including PI3K/Akt, NF-κB, and TGF-β/Smad (Gong, 2025). Meanwhile, astragaloside IV can inhibit the Wnt/β-catenin signaling pathway, degrade the ECM in renal tissue, and improve renal fibrosis (Chen et al., 2024c).

##### 4.2.2.4 The inhibitors of HDAC4

**TMP195.** In the LPS-induced AKI mouse model and the TKPTs treated with LPS, TMP195 improves renal tubule dilation, cell shedding, and renal tubule atrophy induced by LPS through inhibiting HDAC4 expression to exert anti-apoptotic and anti-inflammatory effects. TMP195 can effectively inhibit the expression of HDAC4 in the kidney and increase the level of acetylated H3K14, showing significant epigenetic regulation. TMP195 treatment decreases the expression levels of kidney injury markers NGAL and KIM-1 and inhibits apoptosis by reducing the number of TUNEL-positive cells and cleaved caspase-3-positive tubular cells. Its anti-apoptotic effect is also reflected in restoring the phosphorylation of Bcl-2, inhibiting the expression of Bax-2, and up-regulating the expression of BMP-7. In addition, TMP195 can effectively inhibit the expressions of inflammatory factors ICAM-1, MCP-1, TNF-α, and IL-1β, thereby alleviating kidney inflammation (Zhang W. et al., 2020).

**TMP269.** TMP269, a potent and selective class IIa HDACs inhibitor, significantly improves the pathological changes including renal tubular dilation, cell debris accumulation in the renal tubular lumen, interstitial edema, and inflammatory cell infiltration in FA-/IRI-induced AKI mouse model by reducing the expression of HDAC4 to regulate pathways associated with apoptosis, autophagy, and proliferation. At the molecular level, TMP269 inhibits the increased expressions of HDAC4 and acetyl-histone H3 induced by FA and I/R and reduces the expression of caspase-3 cleavage, Bax, and phosphorylation of p53. At the same time, the expressions of E-cadherin, BMP7, Klotho, and Bcl-2 in the injured kidney are restored (Li J. et al., 2022).

**Magnolol.** In high-fructose cultured human podocytes (HPCs), the upregulation of Sp1 promotes the expression of HDAC4 and leads to the disappearance of the foot process and the intensification of the inflammatory response. Magnolol can significantly inhibit the increase in triokinase/FMN cyclase (TKFC), Sp1 but reduce the combination of Sp1 and HDAC4 to reduce HDAC4 and NICD1 protein levels, further inhibiting the activation of the Notch1 pathway to mitigate podocyte inflammation. Notably, the TMP195 and allopurinol show similar protective effects, significantly reducing TNF-α expression in HPCs exposed to high-fructose diet by inhibiting HDAC4 activity, thereby alleviating inflammatory damage induced by a high-fructose diet (Zhou Z. et al., 2024).

Low concentrations of magnolol can promote the cell survival rate in a dose-dependent manner. However, magnolol can cause toxicity and inhibit the survival of microvascular endothelial cells (MMECs) within the concentration range of 50–200 μg/mL^-1^ (Lin et al., 2021). Magnolol also show the potential to increase the activity or expression of angiotensin-converting enzyme 2 (ACE-2) and thus may aggravate severe acute respiratory syndrome coronavirus 2 (SARS-CoV-2) infection (Junior et al., 2021).

##### 4.2.2.5 The inhibitors of HDAC5

**Trichostatin A.** The expression of HDAC5 is significantly upregulated in a variety of renal disease models, including STZ-induced diabetic mice, UUO mice, and HK-2 cells treated with HG. The overexpression of HDAC5 promotes EMT process, downregulates E-cadherin expression, and upregulates α-SMA expression. However, TSA can effectively downregulate the expression levels of HDAC5 and TGF-β1, while inhibiting the expression of EMT markers α-SMA in RTECs of diabetic mice, thus significantly reducing the pathological deposition of renal ECM in diabetic mice (Xu Z. et al., 2021).

##### 4.2.2.6 The inhibitors of HDAC6

**Trichostatin A.** TSA inhibits the expressions of HDAC6 and HDAC10 in renal tissue to reduce the proliferation of the GBM and matrix, alleviate the thickening of the GBM, and delay the development of DKD in db/db mice and mTECs treated with HG/AGEs. Mechanistically, TSA treatment increases the transcription levels of transcription factor EB (TFEB) and its downstream autophagy-lysosome pathway-related genes, including cystinosin and hermansky-pudlak syndrome 1. Additionally, TSA reduces the apoptosis rate of RTECs by enhancing the acetylation level of TFEB. Notably, the knockout of HDAC6 partially blocks the upregulation of TFEB downstream gene expression induced by TSA, indicating that the effect of TSA on TFEB activation depends on the inhibition of HDAC6 (Li X. et al., 2024).

**2-methylquinazoline derivative F7.** The 2-methylquinazoline derivative F7, a compound that selectively inhibits HDAC6, can significantly alleviate the dilation, swelling, necrosis, and tubular formation of renal tubules and improve acute renal insufficiency in glycerol-induced rhabdomyolysis AKI mouse model and the HK-2 cells treated with ferrous myoglobin. Mechanistically, F7 enhances the acetylation of histone 3 and α-tubulin by reducing the activity of HDAC6, inhibiting the expression of ERK1/2 protein, blocking the phosphorylation process of p38, and regulating the NF-κB signaling pathway. In addition, F7 treatment significantly reduces the expressions of pro-inflammatory factors IL-1β and IL-6 in kidney and HK-2 cells to alleviate inflammatory response and achieve protection against rhabdomyolysis-induced AKI (Liu et al., 2019).

**2-methylquinazoline derivative 23BB.** The 2-methylquinazoline derivative 23BB alleviates the dilation, swelling, and necrosis of renal tubules in cisplatin-induced AKI mice model and the HK-2 cells treated with cisplatin through inhibiting HDAC6 activity. Mechanically, 23BB effectively inhibits the expression and activity of HDAC6 to enhance the acetylation level of histone H3 and α-tubulin. Specifically, 23BB treatment effectively reverses the increase in p-PERK and eIF2α associated with ER stress induced by cisplatin and increases the ATF4 expression, alleviating ER stress. In addition, HDAC6 inhibition also decreases the expressions of pro-inflammatory cytokines IL-1β and IL-6, decreases the number of TUNEL-positive renal tubular cells, and regulates the expression of apoptosis-related proteins, thereby alleviating ER stress-induced apoptosis in cisplatin-stimulated HK-2 cells (Hao et al., 2020).

**CAY10603.** CAY10603 significantly inhibits the activation of the NLRP3 inflammasome in the STZ-induced DKD mice model, HK-2 cells stimulated by TGF-β, and ATP/nigericin-induced iBMDM inflammation model by inhibiting the expression of HDAC6 to reduce the infiltration of macrophages and improve the renal tubule injury and interstitial fibrosis. At the molecular level, CAY10603 inhibits the activation of NLRP3 and caspase-1 induced in HK-2 cells and iBMDM, effectively alleviating NLRP3-mediated macrophage pyroptosis. In addition, CAY10603 also blocks the upregulation of collagen type I alpha 1 chain (Col1a1) and α-SMA expression by inhibiting the expression of HDAC6, improves renal function, and inhibits renal tubulointerstitial fibrosis in STZ-induced DKD mice (Hou et al., 2022).

**Tubacin2.** Tubacin2 can enhance autophagy to mitigate podocyte injury in the kidneys of DKD patients, db/db mice, and podocytes treated with AGE through the inhibitory effect on HDAC6. Specifically, tubacin inhibits the activity and expression of HDAC6, inhibits P62 and LC3-II, and upregulates the expression of beclin-1 (the key regulators of autophagy) by enhancing the acetylation of α-tubulin at lysine 40 residues, thereby restoring autophagy levels and motility in AGE-treated podocytes (Liang et al., 2020).

Tubacin can inhibit cyst growth in canine renal epithelial cells (MDCK.2 cells) and Pkd1-specific knockout ADPKD mouse model. Notably, tubacin shrinks cysts in cells that have already formed cysts by inhibiting the expression of HDAC6. Mechanistically, tubacin increases the acetylation of tubulin and downregulates the expression of cyclic adenosine monophosphate (cAMP, whose elevated expression can promote the formation and development of renal cysts), thus inhibiting the proliferation of MDCK-2 cells and Pkd1-specific knockout ADPKD mouse model. Interestingly, TSA has a similar effect in inhibiting cyst formation in MDCK-2 cells and Pkd1-specific knockout ADPKD mouse in a dose-dependent manner (Cebotaru et al., 2016).

**Compound 26 (benzothiazole derivative).** Compound 26, a novel HDAC inhibitor incorporating a benzothiazole moiety, effectively inhibits the HDAC6 activity to significantly inhibit the formation and expansion of cysts in an MDCK cyst model, embryonic renal cyst model and Pkd1 knockout mouse model. Mechanically, compound 26 enhances the α-tubulin acetylation by inhibiting HDAC6, inhibiting cystic epithelial cell proliferation and cyst enlargement in embryonic renal cyst model and Pkd1 knockout mice (Cao et al., 2024).

##### 4.2.2.7 The inhibitors of HDAC8

**PCI-34051.** PCI-34051 is a highly selective inhibitor of HDAC8. In the cisplatin-induced AKI mouse model and the mouse renal epithelial cells (mRTEC) treated with cisplatin, PCI-34051 significantly reduces tubular damage and apoptosis, alleviates DNA damage and promotes its repair. The PCI-34051 inhibits the expressions of HDAC8 and NGAL to alleviate renal tubular damage. In addition, PCI-34051 reduces the expressions of p53 and p21 and the phosphorylation of CDK2 to inhibit apoptosis and cell cycle arrest. Similarly, the PCI3405 significantly decreases PARP1, caspase-3 cleavage, and Bax/Bcl-2 ratio to reduce apoptosis and increase cell viability. In addition, the inhibition of HDAC8 reverses the increased expression of γ-H2AX, an early marker of DNA double-strand breaks (DSB), and decreased expression of MRE11, a protein associated with DNA homologous recombination (HR) repair, thereby alleviating DNA damage and promoting its repair in kidney and renal epithelial cells (Wang et al., 2024c).

In UUO-induced renal fibrosis mouse model and TKPT cells treated with TGF-β1, PCI34051 alleviates renal interstitial fibrosis by inhibiting HDAC8 expression and activity. Specifically, PCI34051 blocks the activation of TGF-β and p-Smad, reducing the phosphorylation of STAT3 and β-catenin in renal tubular cells, and reversing the increase in HDAC8 expression, the increased deacetylation levels of cortical protein and the expression of fibrosis markers (α-SMA, collagen 1, and fibronectin) induced by UUO. PCI-34051 also inhibits the expression of phospho-histone 3 at serine 10 and reduces the number of tubular epithelial cells that remained in the G2/M phase of the cell cycle (Zhang Y. et al., 2020).

##### 4.2.2.8 The inhibitors of HDAC9

**TMP-195.** In the AAN/UUO-induced renal fibrosis mouse model and the HK-2/NRK-49F cells treated with AAN, TMP-195 effectively inhibits the expression of HDAC9, attenuates epithelial cell cycle arrest in the G2/M phase, and then reduces the production of pro-fibrotic cytokines and alleviates renal tubulointerstitial fibrosis in mice. Mechanistically, the use of TMP-195 interferes with the interaction between HDAC9 and STAT1, reduces the deacetylation level of STAT1 in HK-2 cells, and prevents its phosphorylation and reactivation, thereby inhibiting G2/M phase arrest of TECs and alleviating renal tubulointerstitial fibrosis (Zhang et al., 2023c).

##### 4.2.2.9 The inhibitors of HDAC11

**Quisinostat.** Quisinostat mitigates renal fibrosis in the RTECs of the UUO-induced renal fibrosis mouse model and Ang II-treated HK-2 cells. Mechanistically, quisinostat significantly downregulates the expression of HDAC11 and reduces the expression of pro-fibrotic factors and pro-inflammatory mediators to inhibit renal fibrosis and inflammation induced by UUO/Ang II. Specifically, treatment with quisinostat inhibits the expression of HDAC11 and reduces the interaction between HDAC11 and activator protein 2 (AP-2α) on the KLF15 promoter to promote KLF15 transcription and upregulate KLF15 expression, thereby relieving renal fibrosis induced by UUO and Ang II (Mao et al., 2020)

## 5 Conclusion and perspectives

Acetylation modification is a key PTM that is dynamically regulated by HATs and HDACs. The modification is widely involved in cell metabolism, proliferation, differentiation, and apoptosis by affecting protein stability, activity, subcellular localization, and protein interaction (Dang and Wei, 2022). Acetylation modification also plays an important role in maintaining healthy homeostasis of the kidneys by finely regulating the inflammatory response, oxidative stress response, apoptosis process, and fibrotic process (Jiang et al., 2023).

AKI is a clinical syndrome of rapid decline in renal function, and the primary causes of AKI encompass infections and ischemia, sepsis, medication or invasive procedures. Long-term complications of AKI include CKD, renal failure, and cardiovascular diseases, with an increased risk of mortality (Hoste et al., 2018; Kellum et al., 2021; Ostermann et al., 2025). CKD encompasses a variety of conditions, including DKD, HTN, and PKD (Tang et al., 2020; Kalantar-Zadeh et al., 2021). DKD is chronic kidney damage caused by diabetes, characterized by proteinuria and progressive decline in renal function, and is the main cause of ESRD (Hu et al., 2024). HTN is caused by chronic uncontrolled hypertension and is characterized by glomerular sclerosis and decreased kidney function (Dikalov and Kirabo, 2023). PKD is a genetic kidney disease characterized by multiple cysts on both kidneys, ultimately leading to loss of kidney function (Bergmann et al., 2018). The common feature of these kidney diseases is that they can all eventually develop ESRD, but disease progression can be delayed by early intervention.

**FIGURE 3:**
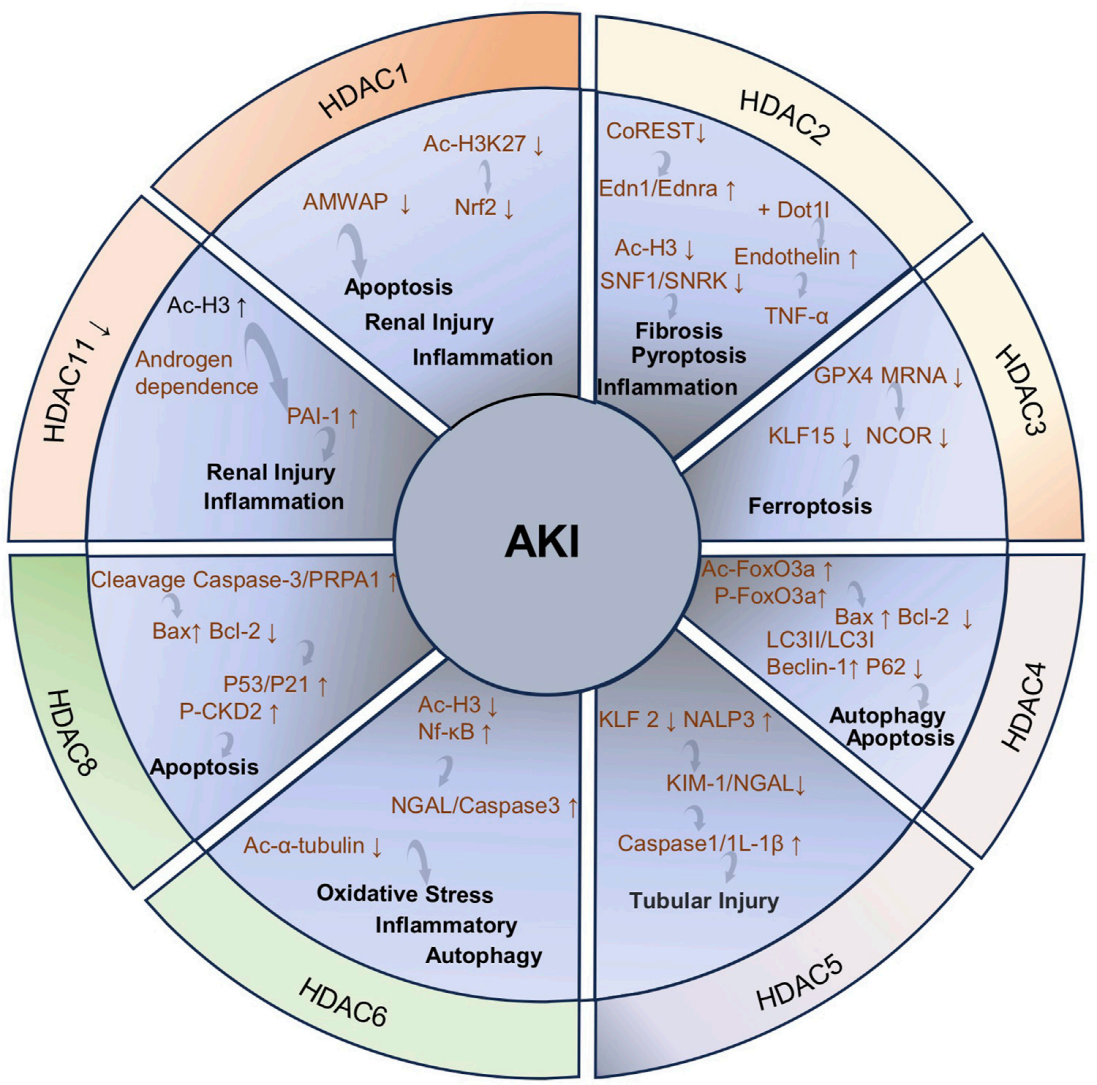
The mechanism of HDACs in AKI.

**FIGURE 4:**
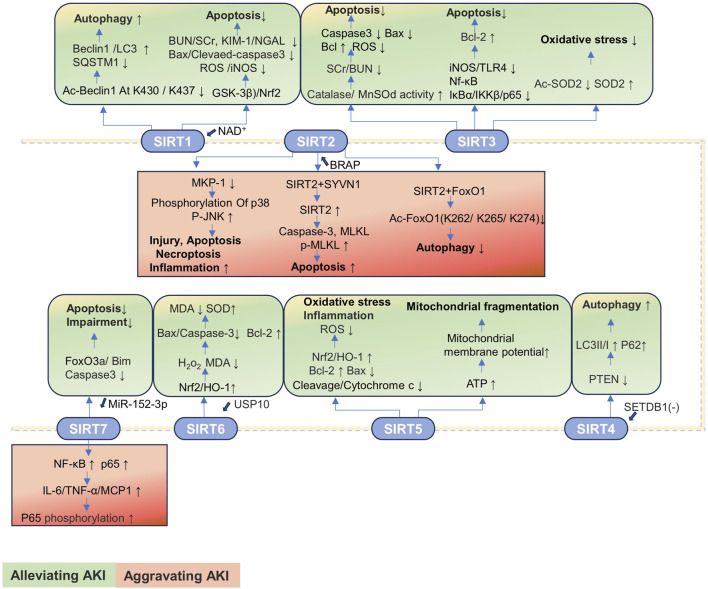
The mechanism of HDACs in DKD.

**FIGURE 5:**
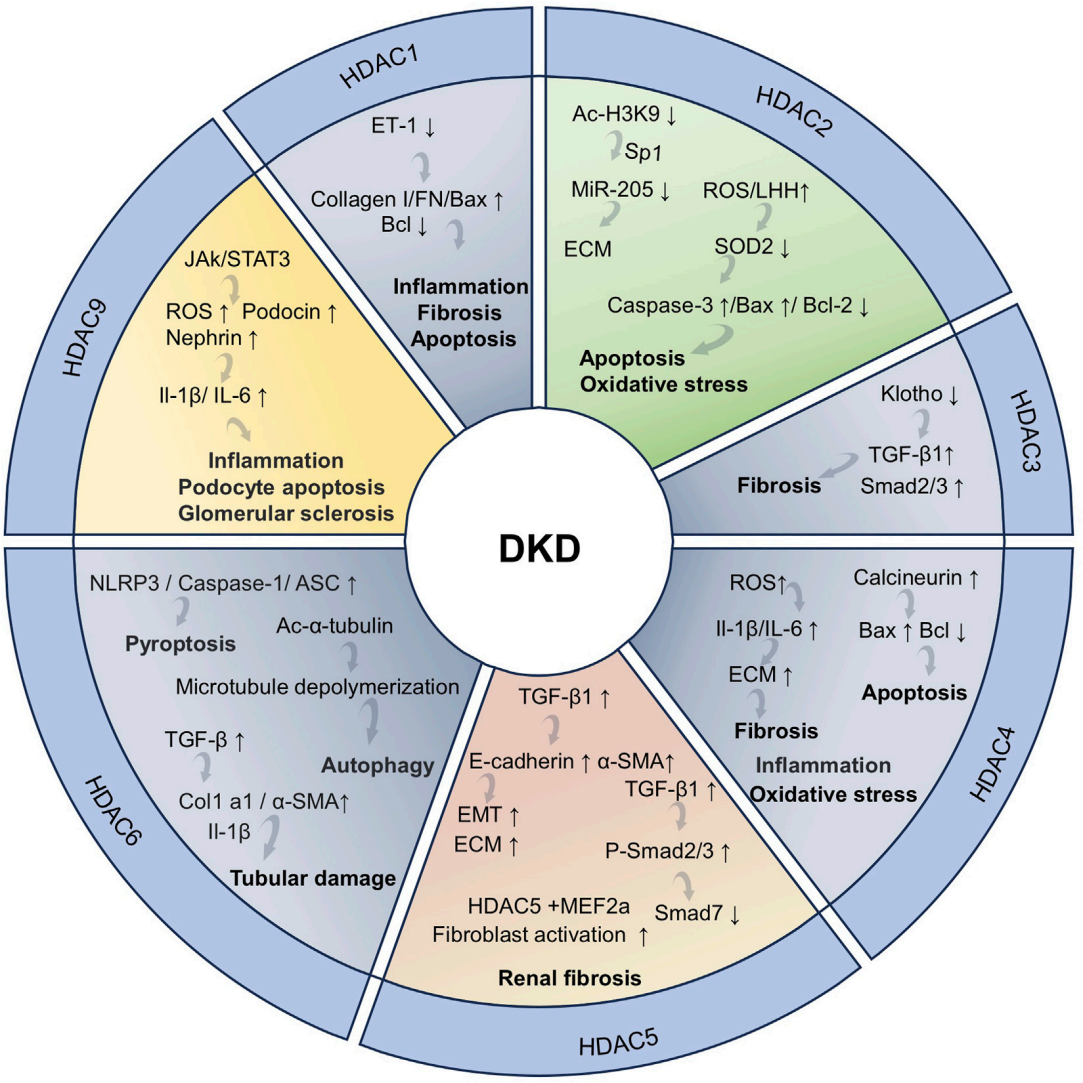
The mechanism of SIRTs in AKI.

**FIGURE 6:**
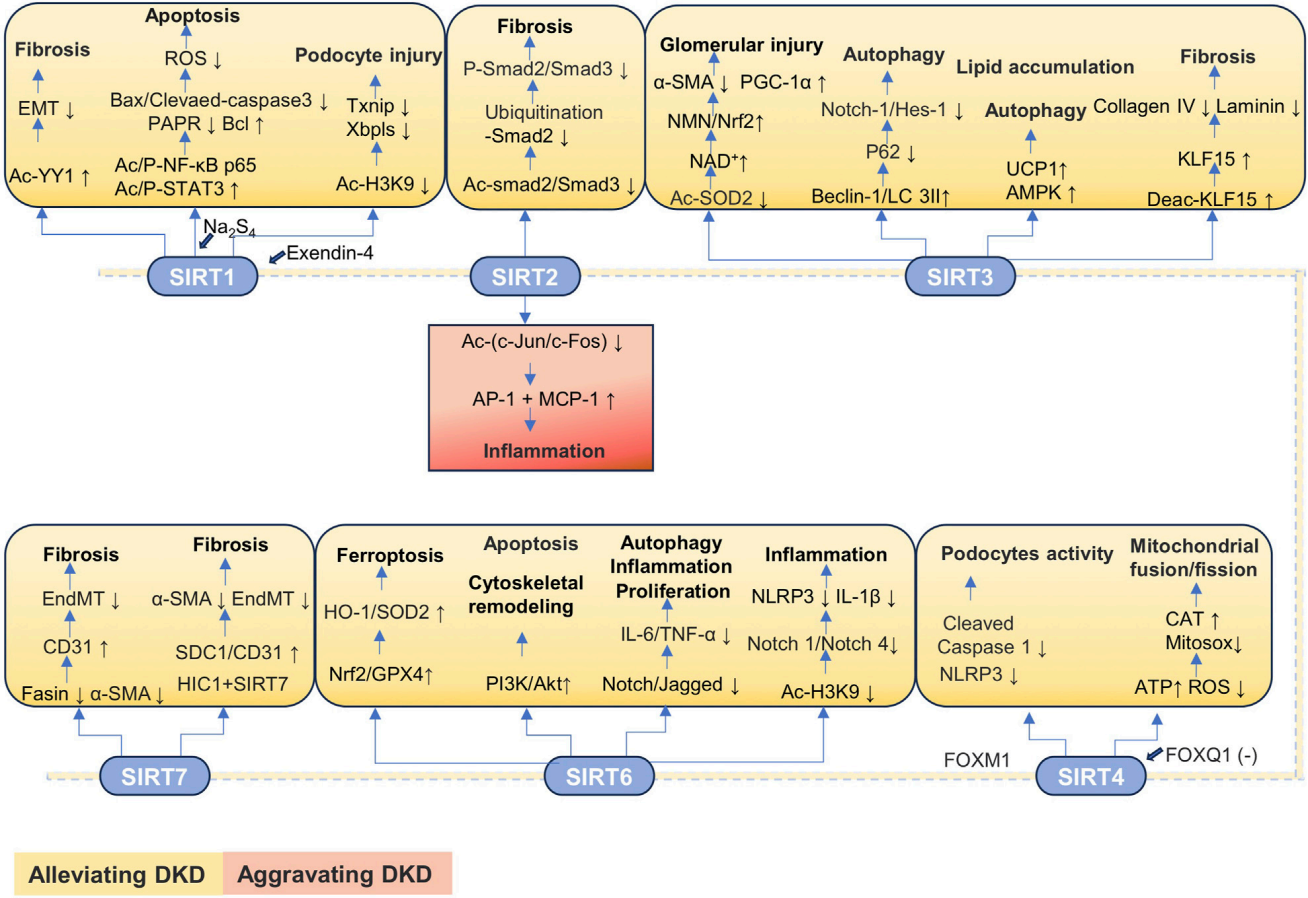
The mechanism of SIRTs in DKD.

**FIGURE 7:**
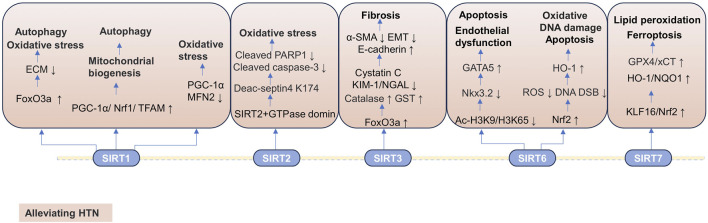
The mechanism of SIRTs in HTN.

HDACs play a key role in the occurrence and development of the kidney disease (including AKI, DKD, HTN, and PKD) by increasing the deacetylation level of key proteins, regulating inflammation response, fibrosis, apoptosis, interacting with the endothelin system, and cell proliferation (Figure 3; Figure 4). Many inhibitors targeting specific HDAC subtypes and HDAC pan-inhibitors have been shown to have renoprotective effects and may be potential strategies for future kidney disease treatment (Bush and McKinsey, 2010. The expression and activity of different SIRT subtypes in kidney disease are different and are closely related to the type of injury. Most SIRTs can play a core protective role in various renal diseases through antioxidant, mitochondrial protection, anti-inflammatory, immune regulation, anti-apoptosis, and autophagy regulation (Figure 5; Figure 6; Figure 7).

Existing studies on HDAC7, HDAC10, and HDAC11 in kidney disease are relatively lacking. In particular, the HDAC11, as a newly discovered HDAC, has very limited studies on its role and mechanism in kidney disease. Although no current studies have confirmed the direct role of HDAC7, HDAC10, and HDAC11 in nephropathy, some studies have shown that HDAC7 activates the MAPK/NF-κB signaling cascade and subsequent gene transcription, enhances the expression of pro-inflammatory cytokines, thereby intensifying the inflammatory response in LPS-stimulated macrophages and mouse model through directly interacting with the complex of TNF receptor-associated factor 6 and TGFβ -activated kinase 1 (TRAF6-TAK1) (Kadier et al., 2024). Therefore, regulating the inhibition of the MAPK/NF-κB signaling pathway by suppressing the expression and activity of HDAC7 may also become a therapeutic strategy for LPS-induced nephropathy. HDAC10 has been proven to promote airway inflammation in asthma by interacting with STAT3 to control macrophage polarization (Zhong et al., 2023). However, another study has shown that HDAC10 inhibits the NF-κB signaling pathway and the expression of inflammatory factors TNF-α and IL-6 in silicosis to mitigate inflammation. Furthermore, HDAC10 has been proven to reduce ROS, alleviate oxidative stress, and reduce pulmonary fibrosis (Tian et al., 2023). HDAC11, as the newly discovered member of the HDACs family, has also been rarely studied in kidney diseases. However, one study has confirmed the important role of HDAC11 in the development of renal fibrosis. The inhibition of HDAC11 restores histone acetylation around the KLF15 promoter to alleviate the fibrotic process in a renal fibrosis model induced by UUO/HFD/Ang II (Mao et al., 2020). As for the role and mechanism of the SIRT family in kidney disease, current studies mainly focus on SIRT1, SIRT3, and SIRT6.

Notably, although most SIRTs play a protective role in kidney diseases, some SIRTs have been confirmed to exacerbate renal disease induced by multiple causes, while pharmacological inhibition or gene knockout of these SIRTs can exert renoprotection (Jung et al., 2020; Chen G. et al., 2022; Sánchez-Navarro et al., 2022; Yu et al., 2025). For example, the overexpression of SIRT1 can decrease the acetylation level of retinoblastoma protein (Rb) in Pkd1-deficient MEK cells but increase its phosphorylation level, regulating the proliferation signal of renal cystic epithelial cells, and accelerate the growth of cysts in ADPKD patients (Zhou et al., 2013). In the context of AKI, the overexpression of SIRT2 enhances the phosphorylation of p38 and JNK in renal and tubular epithelial cells to deteriorate cisplatin-induced tubular damage, inflammation, and aggravate renal dysfunction. The knockout of SIRT2 can reverse these processes (Jung et al., 2020). Additionally, the overexpression of SIRT2 increases the deacetylation level of FoxO1 to inhibit autophagy in HK-2 cells treated with LPS and increases the production of pro-inflammatory factors, thereby aggravating CLP-induced SAKI (Yu et al., 2025). SIRT7 plays a dual role in AKI, which can both exert a protective effect and may also promote damage under certain conditions. In the context of I/R-induced AKI, the increased SIRT7 expression by upregulating miR-152-3p reduces the expression of exogenous apoptosis molecules and the apoptosis of renal cells, mitigating I/R-induced apoptosis and functional impairment of renal cells (Wang et al., 2023d). Interestingly, another study confirmed that the SIRT7 can aggravate kidney damage in I/R-induced AKI, and the knockout of SIRT7 protects mice from I/R-induced AKI through inhibiting the activation of NF-κB pathway and reducing the expression of pro-inflammatory factors. Additionally, the inhibition of SIRT7 and NF-κB signaling pathway prevents apoptosis to alleviate AKI induced by cisplatin (Chen G. et al., 2022; Sánchez-Navarro et al., 2022). Notably, in the context of DKD, SIRT2 has a dual role. SIRT2 enhances the deacetylation of Smad2 at Lys451 and Smad3 at Lys341 and Lys378 to inhibit the activation of Smad2 and Smad3 and mitigate kidney injury in a UUO mice model and DKD patients (Yang et al., 2023). However, SIRT2 reduces cell proliferation and apoptosis by reducing the level of autophagy in glomerular podocytes to deteriorate DKD (Liu S. et al., 2022). Additionally, SIRT2 increases the deacetylation level of c-Jun/c-Fos and increases the binding activity of AP-1 and the inflammatory factor MCP-1, thereby aggravating the HG/PA-induced inflammatory response (Chen et al., 2025). The different roles of SIRT2/SIRT7 in DKD/AKI may be related to the injury stage, cell type or crosstalk with other signaling molecules.

Although many agonists or inhibitors have shown significant efficacy for kidney diseases in preclinical studies, the use of most agonists and inhibitors in kidney diseases remains controversial (Hyndman, 2020; Huang et al., 2022; Zheng et al., 2025). The controversy over the application of agonists and inhibitors mainly lies in three aspects: off-target risk, dose dependence, and insufficient clinical data.

At present, most of the extensively studied HDAC inhibitors or SIRT agonists have broad-spectrum effects; these inhibitors or activators can affect multiple HDAC subtypes or SIRT subtypes. It is known that the effects of different HDACs or SIRTs are related to multiple factors, including disease type, disease development stage, and the degree of inhibition or excitation by HDACs/SIRTs. For instance, TSA is a class I/II HDAC inhibitors that can target multiple HDAC subtypes (including HDAC1, HDAC2, HDAC3, HDAC5, and HDAC6) and play a protective role in multiple kidney diseases (Table 4). Furthermore, the regulatory effect of the same inhibitor on the same deacetylase may vary with the disease context. For instance, metformin can activate SIRT1 and regulate related pathways to mitigate LPS-induced SAKI and improve renal function of HFD-/STZ-induced diabetic rats (Xu et al., 2020; Zhang W. L. et al., 2024). However, metformin elevates the miR-34a levels to inhibit the SIRT1 levels by directly targeting SIRT1 3 ′-UTR to recover podocyte viability and inhibit podocyte apoptosis in STZ-induced diabetic mice and HG-treated podocytes (Zhuang et al., 2024). Therefore, the regulation of SIRT1 by metformin shows disease background dependence, thereby exerting different protective effects. This difference in effect may be related to the energy metabolism status, oxidative stress level, inflammatory signaling pathways, and the upstream/downstream targets of SIRT1. It is worth noting that pan-agonists/pan-inhibitors like metformin, due to their broad-spectrum inhibitory properties, may interfere with nontarget epigenetic regulatory pathways (such as transcription factors and non-histone acetylation modifications), thereby increasing the risk of off-target. Furthermore, current research on broad-spectrum agonists or inhibitors mostly focuses on one or two targets, lacking in-depth exploration of the crosstalk produced when the same agonist/inhibitor acts on multiple members of the SIRT/HDAC family. Meanwhile, how to enable pan-inhibitors such as TSA or VPA to precisely target specific HDACs and influence the progression of kidney diseases by regulating their activity or expression is also a key issue that needs to be urgently addressed.

**TABLE 5:** The clinical trial schedule for agonists and inhibitors.

Agonists/inhibitors		Drug	Test topic	Indication	Region	Stage	Registration number
Agonists	SIRTs	Metformin	Implementation of Metformin theraPy to Ease DEcline of kidney function in Polycystic Kidney Disease (IMPEDE-PKD): randomised placebo-controlled trial	PKD	UK	3	ISRCTN12436830
			A clinical trial was conducted to test and study the absorption, distribution, and metabolism of the drug ANJ900 in healthy Chinese populations, as well as the impact of food on these conditions	CKD	CN	1	CTR20212265
		Quercetin	Senescence in chronic kidney disease	CKD	USA	2	NCT02848131
		Melatonin	Evaluation of effect of melatonin in treatment of acute kidney injury	AKI	Iran	3	IRCT20250110064339N1
			Evaluation of the melatonin effect on proteinuria, inflammatory and oxidative stress factors in patients with chronic kidney disease	CKD	Iran	3	IRCT20160412027346N12
			The effect of melatonin and atorvastatin on diabetic nephropathy	DKD	Iran	3	IRCT20181118041689N1
Inhibitors	SIRTs	Berberine	Berberine prevent contrast-induced nephropathy in patients with diabetes	CKD	CN	4	NCT02808351
			A prospective, single-center, randomized controlled study of the efficacy of oral berberine hydrochloride in the treatment of diabetic nephropathy	DKD	CN	2	ChiCTR2400083889
	HDACs	Rutin	Evaluation of the effect of rutin and vitamin c on the oxidative stress in hemodialysis patients	ESRD	Egypt	3	NCT04955145
		Astragaloside	Effect of Astragalus membranaceus (Fisch.) Bunge extract containing Astragaloside IV on renal function and proteinuria in patients with diabetic nephropathy	DKD	Myanmar	1/2	TCTR20200305003

Although there is currently a lack of definite clinical trial data to prove that the efficacy of SIRT agonists or HDAC inhibitors has a clear dose–response relationship with their dosage, research studies have demonstrated that the nephroprotective effects of resveratrol exhibit dose-dependent characteristics. At lower doses (≥25 mg/kg), resveratrol can improve renal function, whereas higher doses (≥50 mg/kg) paradoxically exacerbate renal fibrosis (Wang R. et al., 2022).

Studies have shown that the improper dosage may cause some adverse reactions. During the treatment of acute diseases, metformin may increase the risk of AKI, lead to a decline in renal function, and increase the risk of complications and death. For the use of metformin, most pharmacists recommend adjusting the dosage to reduce potential risks (Truong et al., 2024). Additionally, the high dose of the honokiol microemulsion induces developmental toxicity in rats and zebrafish by inducing oxidative stress (Li H. et al., 2022). In addition, excessive intake of VPA may cause central nervous system depression, and it can lead to coma, multiple organ failure, and death in severe cases (Zaringhalam et al., 2025). Although VPA has been proven in preclinical studies to alleviate renal injury and improve renal function by inhibiting the activity of multiple HDAC subtypes, VPA may increase ROS and lead to renal injury (Alhusaini et al., 2024). Relevant studies have shown that MG is cytotoxic. Low concentrations of MG can promote cell survival rate in a dose-dependent manner. When the concentration is below 60 μM, MG can promote the survival of U937 cells. When exposed to MG at a concentration lower than 70 μM after 48 h, the mortality rate of LO-2 cells was less than 20%. Furthermore, within the concentration range of 50–200 μg mL^-1^, MG can cause toxicity and inhibit the survival of microvascular endothelial cells (MMECs) (Lin et al., 2021). Magnolol also show the potential to increase the activity or expression of ACE-2, and thus may aggravate SARS-CoV-2 infection (Junior et al., 2021).

Although many agonists or inhibitors have shown significant efficacy in preclinical studies for kidney diseases, the research on HDAC inhibitors and SIRT agonists in tumors (such as uncontrolled proliferation caused by abnormal deacetylation modifications) is more extensive. For instance, TSA and VPA, as classic broad-spectrum HDAC inhibitors, are widely used in basic cancer research, but their research in kidney diseases is very limited. In addition, a large number of preclinical studies have shown that astragaloside I has significant anti-tumor efficacy in various malignant tumor models such as renal cell carcinoma, gastric cancer, colorectal cancer, breast cancer, and non-small cell lung cancer. Its mechanism involves the regulation of key signaling pathways such as PI3K/Akt and NF-κB (Hu C. et al., 2025; Ran et al., 2025; Wang Z. et al., 2025; Yang Y. et al., 2025). Magnolol has the significant anti-proliferative, pro-apoptotic, and anti-metastasis effects in various malignant tumor models (Chen et al., 2024b; Cheng et al., 2025; Gao et al., 2025; Wang et al., 2025d). VPA is currently often used in combination with other drugs in clinical practice for various cancers, including breast cancer, cervical cancer, and prostate cancer. At present, most of the research on these inhibitors/agonists in the field of kidney diseases is still limited to the preclinical stage (Figure 5). Their potential efficacy has only been verified through *in vitro* cell models and animal experiments. Their application in kidney diseases still faces challenges including low selectivity, strong side effects, and insufficient delivery efficiency. Therefore, this increases the uncertainty of SIRTs and HDAC targeted agents in clinical applications.

In conclusion, this study reviews the significant role of acetylation and deacetylation in renal physiology and pathology and focuses on introducing the roles and mechanisms of deacetylases HDACs and SIRTs, which regulate the deacetylation process in renal physiology and pathology. We discuss the dual roles of the SIRT family in renal diseases. In addition, we also discuss the HDAC inhibitors and SIRT agonists with renal protective effects and their mechanisms of action.
